# Identification of qPCR reference genes suitable for normalizing gene expression in the *mdx* mouse model of Duchenne muscular dystrophy

**DOI:** 10.1371/journal.pone.0211384

**Published:** 2019-01-30

**Authors:** John C. W. Hildyard, Amber M. Finch, Dominic J. Wells

**Affiliations:** Department of Comparative Biomedical Sciences, Royal Veterinary College, London, United Kingdom; University of Minnesota Medical Center, UNITED STATES

## Abstract

The *mdx* mouse is the most widely-used animal model of the human disease Duchenne muscular dystrophy, and quantitative PCR analysis of gene expression in the muscles of this animal plays a key role in the study of pathogenesis and disease progression and in evaluation of potential therapeutic interventions. Normalization to appropriate stably-expressed reference genes is essential for accurate quantitative measurement, but determination of such genes is challenging: healthy and dystrophic muscles present very different transcriptional environments, further altering with disease progression and muscle use, raising the possibility that no single gene or combination of genes may be stable under all experimental comparative scenarios. Despite the pedigree of this animal model, this problem remains unaddressed. The aim of this work was therefore to comprehensively assess reference gene suitability in the muscles of healthy and dystrophic mice, identifying reference genes appropriate for specific experimental comparisons, and determining whether an essentially universally-applicable set of genes exists. Using a large sample collection comprising multiple muscles (including the tibialis anterior, diaphragm and heart muscles) taken from healthy and *mdx* mice at three disease-relevant ages, and a panel of sixteen candidate reference genes (*FBXO38*, *FBXW2*, *MON2*, *ZFP91*, *HTATSF1*, *GAPDH*, *ACTB*, *18S*, *CDC40*, *SDHA*, *RPL13a*, *CSNK2A2*, *AP3D1*, *PAK1IP1*, *B2M and HPRT1*), we used the geNorm, BestKeeper and Normfinder algorithms to identify genes that were stable under multiple possible comparative scenarios. We reveal that no single gene is stable under all conditions, but a normalization factor derived from multiple genes (*RPL13a*, *CSNK2A2*, *AP3D1* and the widely-used *ACTB*) appears suitable for normalizing gene expression in both healthy and dystrophic mouse muscle regardless of muscle type or animal age. We further show that other popular reference genes, including *GAPDH*, are markedly disease- or muscle-type correlated. This study demonstrates the importance of empirical reference gene identification, and should serve as a valuable resource for investigators wishing to study gene expression in *mdx* mice.

## Background

The X-linked muscle-wasting disease Duchenne muscular dystrophy (DMD) affects roughly 1 in 5000 new born boys [1], and is the most common fatal genetic condition diagnosed in childhood. Caused by absence or insufficiency of the muscle membrane-associated protein dystrophin, muscle fibres lacking this protein sustain damage under even normal use [2]. Repeated cycles of muscle degeneration and compensatory regeneration, alongside a steady accumulation of fibrotic scarring and fatty replacement, lead to muscle atrophy, loss of function and ultimately death. While the condition is presently incurable, DMD remains a field of active research: several different approaches aimed at dystrophin restoration are currently under investigation or entering therapeutic trials [3, 4]. Such research is aided by animal models, and multiple models of this disease exist, including mouse [5], rat [6, 7], rabbit [8], dog [9–11] and pig [12, 13]. While mouse models (particularly the *mdx* mouse) are the most frequently studied, each model offers discrete benefits and caveats in terms of disease severity, disease progression, cost and therapeutic tractability [14]. Regardless of species, assessment of the consequences of insufficient dystrophin -and more critically, the extent of therapeutic dystrophin restoration- utilises multiple investigative avenues, from whole animal and whole muscle physiological studies, to gross histology and immunostaining, to quantitative measures of gene expression at the protein and mRNA level.

Quantitative analysis of mRNA via reverse-transcription and subsequent qPCR (SYBR green, Taqman, and more recently digital droplet) is a powerful approach, allowing determination of the extent and significance of changes in transcription (or degradation), often needing only minimal starting material. Processing of tissue to RNA and then onward to cDNA involves multiple manipulation steps, however, all of which may vary in efficiency: normalization of data via internal reference genes (genes known to be stably-expressed under the conditions studied) is essential before meaningful conclusions can be drawn. Identification of such reference genes is thus critical, but it is now well-recognised that no single gene is suitable under all circumstances, and many putative reference genes show prominent changes between tissues and -of particular relevance to DMD research- with disease. Different muscles within even the same model organism may necessitate different reference genes, hampering comparability of experiments. An ideal panel of reference genes would be appropriate regardless of muscle group studied, animal age, or whether the muscle in question was healthy or dystrophic, but identifying such a panel presents considerable challenges. Healthy skeletal muscle is essentially static, and consequently exhibits remarkable transcriptional stability. In contrast, the cellular environment present within damaged, regenerating muscle is more diverse, comprising necrotic/apoptotic myofibres, proliferating myoblasts, fusing myotubes and immature myofibres committing to fibre-type fate choices [15]. Add to this the presence of adipocyte, fibroblast, infiltrating macrophage and lymphocyte components characteristic of dystrophic disease progression and it is clear that the transcriptional milieu of dystrophic muscle is likely to be highly plastic. Furthermore, the relative proportions and transcriptional states of all these diverse elements will vary with disease progression, severity and muscle type: finding even a single gene expressed stably under such mixed conditions seems ambitious, and it would not be unreasonable to surmise no such genes exist.

Assessment of potential reference genes is not a straightforward process: RNA is an inherently labile, short-lived molecule, and even within highly-expressed near-ubiquitous mRNAs, transcript levels can alter far more rapidly than the corresponding proteins they encode. *GAPDH* may serve for normalizing protein expression under many conditions, but this gene may conversely be wholly inappropriate as an RNA standard (even under the same conditions) and worse: may be inappropriate in a manner not immediately obvious to the researcher. Poor normalization may mask genuine biological effects, or even suggest such effects where none exist. The importance of reference gene selection in qPCR (and the pitfalls of inappropriate choices) is illustrated in the MIQE guidelines [16]: all qPCR data should be normalized to reference genes shown to be appropriate for the conditions studied, and moreover multiple reference genes should be used (two at a bare minimum). Empirical determination of appropriate genes is by necessity rather tortuous: a simple approach to identify appropriate candidate reference genes would be direct comparison with established good candidates, however such an approach presupposes such candidates exist, and indeed raises questions as to the validation of those established candidates. Identifying suitable reference gene candidates *de novo* thus typically involves generation of a dataset of gene expression values for multiple candidate reference genes, in multiple representative cDNAs covering the expected range of sample variability: this dataset can then be analysed to determine the most stable candidates according to specific comparative metrics. Three commonly-used algorithms are geNorm [17], Bestkeeper [18] and Normfinder [19]: each runs under a Windows/Excel environment (as an executable macro, a write-protected spreadsheet and an Add-In, respectively), rendering them highly accessible to the researcher, and all three can utilise the same essential dataset. Each assesses suitability via subtly different criteria, thus each possesses unique strengths and weaknesses: both geNorm and BestKeeper use pairwise, correlation-based approaches within the dataset, while Normfinder assesses expression stability of individual candidates (download locations and more detailed summaries are provided in S1 Appendix and previously [20]). Strong performance under any single method is commonly adjudged to be sufficient, but strong performance under all three algorithms constitutes firm support for the suitability of any given reference genes. Indeed, combinations of these approaches have been used to determine appropriate reference genes in multiple tissues, disease states and model organisms ranging across the biological spectrum (see [21–32] for a small selection of recent examples). We also note that RefFinder, a software package incorporating all three plus a comparative ΔΔCt method is now available [33], however given the strengths and weaknesses of each method, we prefer to assess outputs individually.

We have previously used the three algorithms described above to identify the genes *CSNK2A2* and *AP3D1* as suitable reference genes throughout the transcriptionally-plastic process of myogenic differentiation in both healthy and dystrophic murine myoblast cell cultures [34], suggesting that such diverse transcriptional environments are tractable to some extent. More recently, we made the remarkable finding that the genes *HPRT1*, *SDHA* and *RPL13a* are appropriate for normalizing gene expression in a canine model of DMD (the DeltaE50-MD dog), regardless of animal age, presence/progression of disease, or even muscle type [20]. A similar panel of genes might thus exist for human patients, or as we investigate here, the *mdx* mouse.

The *mdx* mouse is the canonical animal model of DMD: first reported in 1984 [5], this mouse model carries a natural mutation in exon 23 of the dystrophin gene (*dmd*) which results in a premature stop codon and consequent loss of dystrophin expression [35]. While the disease severity in this animal is mild and remarkably well-tolerated (*mdx* mice live near-normal lifespans, and predominantly display compensatory muscle hypertrophy rather than atrophy and fibrosis) [36], the model still exhibits continual muscle degeneration/regeneration, and has proven to be a valuable resource for scientific research into this devastating human disease.

Given the ubiquity of this animal model (PubMed indicates an average of 124 papers a year since 2000, and ~3000 in total since the model was first reported), it is somewhat surprising that no such reference gene assessment study has to date (to our knowledge) been performed in this animal. Studies have been performed in healthy mice alone, either looking at quadriceps muscle only [37], or at gastrocnemius muscle during denervation atrophy [38], but systematic analysis of candidate reference genes in healthy and dystrophic mice is lacking. A number of different genes have been used in the *mdx* literature, including *B2M* [39], cyclophilin [40] and *HPRT1* [41, 42], but the most widely-employed references appear to be either *GAPDH* [43–47], *18S* [48–50] or *ACTB* [47, 51–53]. In the majority of cases only a single gene is used, with little effort to standardise between studies, and the justification for the reference gene chosen is rarely provided. These shortcomings are perhaps understandable, as identifying and verifying a universally-applicable set of genes is not trivial: assessing the suitability of a given reference gene under multiple criteria necessitates a large and well-structured dataset, one from which statistically-valid criterion-specific subsets can readily be prepared and analysed.

**Fig 1:**
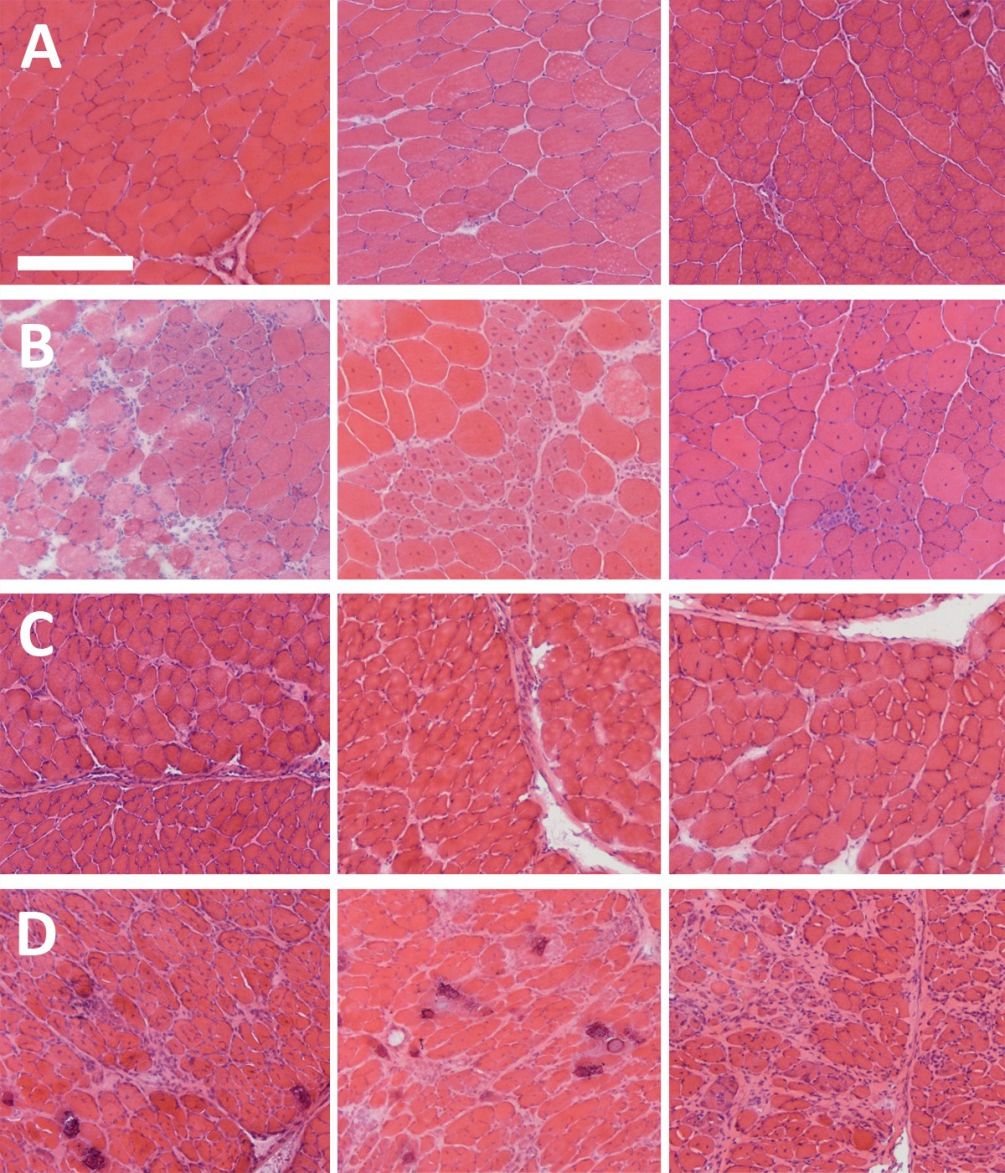
Disease progression in the *mdx* mouse. Representative Haematoxylin and Eosin stains of tissues used in this study. Top row (**A**): WT *Tibialis anterior* (TA) muscle; Second row (**B**): *mdx* TA muscle; Third row (**C**): WT diaphragm muscle; Bottom row (**D**): *mdx* diaphragm muscle. Left column: 6 week samples; Centre column: 10 week samples, Right column: 24 week samples. Note the prominent necrosis and oedema characteristic of the 6-week acute phase, the steady accumulation of centrally-nucleated fibres with age and the marked fibrosis and calcium deposition in dystrophic diaphragms. Scale bar: 200um.

For the study described here, we were able to take advantage of the higher sample numbers mouse models potentially offer, and thus endeavoured to prepare a broad and comprehensive sample set of healthy and *mdx* mouse muscle tissues. Our final sample set comprised muscle RNA collected from dystrophic mice and healthy strain-matched controls at three different disease-relevant ages: 6 weeks, corresponding to the early stage of disease and characterised by profound focal degeneration; 10 weeks, corresponding to progressing disease, with widespread degeneration and compensatory regeneration; and 24 weeks, corresponding to established disease with a comparatively stable level of degeneration/regeneration and fibrotic accumulation in some muscles (Fig 1). We used multiple mice per age (N = 3 per genotype) and a broad panel of muscles covering multiple functional roles, oxidative capacities and dystrophic progression: the highly-studied tibialis anterior (TA) muscle; the gastrocnemius (GC) and quadriceps (Q) hind-limb muscles; the triceps muscle (TRI); musculature of the body wall (BW); the diaphragm (one of the few muscles in the *mdx* mouse to show pronounced fibrotic accumulation); and finally the heart. This substantial panel of samples (126 unique cDNA preparations, see S2 Appendix for a summary) permits empirical assessment of gene stability under a multitude of different criteria or subdivisions. Similarly, we used sixteen candidate reference genes (*FBXO38*, *FBXW2*, *MON2*, *ZFP91*, *HTATSF1*, *GAPDH*, *ACTB*, *18S*, *CDC40*, *HPRT1*, *SDHA*, *RPL13a*, *CSNK2A2*, *AP3D1*, *PAK1IP1* and *B2M* –see Table 1), an extensive panel that included the three most prevalent ‘housekeeping genes’ in the *mdx* field (*18S*, *GAPDH* and *ACTB*), those that scored highly in our murine cell-culture myogenesis work (*CSNK2A2*, *AP3D1*) [34], and those that performed well in our recent dystrophic dog study (*HPRT1*, *SDHA*, *RPL13a*) [20].

**Table 1:** Candidate reference gene names/full names.

Gene Name	Full name
FBXO38	F-Box Only Protein 38
FBXW2	F-Box And WD-40 Domain Protein 2
MON2	Regulator Of Endosome-To-Golgi Trafficking
ZFP91	Zinc Finger Protein 91
HTATSF1	HIV-1 Tat Specific Factor 1
GAPDH	Glyceraldehyde-3-Phosphate Dehydrogenase
ACTB	Beta Actin
18S	18S ribosomal RNA
CDC40	Cell Division Cycle 40
HPRT1	Hypoxanthine phosphoribosyltransferase 1
SDHA	Succinate dehydrogenase subunit A
RPL13a	Ribosomal protein L13
CSNK2A2	Casein Kinase 2 Alpha 2
AP3D1	Adaptor-Related Protein Complex 3 Subunit Delta-1
PAK1IP1	P21-Activated Protein Kinase-Interacting Protein 1
B2M	Beta 2 Microglobulin

We reasoned that use of such a large panel of candidates would maximise potential for identifying reference genes suited for specific comparative scenarios and increase likelihood of identifying a unified set that score highly under all scenarios. As shown below, we were able to determine just such a unified set, while also revealing stark transcriptional differences between model systems of DMD and identifying several widely-used reference genes as being actively muscle- or disease-associated.

## Methods

### Animals and tissue collection

Dystrophic muscle samples were obtained from male *mdx* mice bred under UK Home Office Project Licence PPL 70/7777 (holder Professor Dominic Wells) together with healthy strain-matched male C57/Bl10 wild type (WT) samples from mice bred under the same licence. This study was internally reviewed and approved by the Royal Veterinary College Animal Welfare and Ethical Review Body. Mice were held in open top cages in a minimal disease unit at an average 21°C in a 12 hours light/ 12 hours dark light cycle with food and water provided ad-lib. Mice were collected at three ages (6 weeks, 10 weeks, 24 weeks) and sacrificed by cervical dislocation. Three healthy and three dystrophic animals were used for each time point. Muscle tissues -TA, GC, Q, TRI, BW, hemidiaphragm- were collected rapidly, flash frozen under liquid nitrogen and stored at -80°C. Hearts, contralateral muscles and the remaining hemidiaphragm were mounted in cryoMbed (Bright Instruments Ltd.) on cork blocks and frozen in liquid-nitrogen-cooled isopentane for cryosectioning and histological analysis.

### RNA isolation

Frozen tissues were pulverised under liquid nitrogen while cork-mounted hearts were subjected to cryosectioning. Interleaving unmounted heart sections (~50 sections at 8um thickness) and pulverised muscle tissue (~100mg per sample) were used to prepare RNA via TRIzol reagent (Invitrogen) according to the manufacturer’s instructions (with the addition of a 1:1 chloroform extraction step after phase separation, and inclusion of 10ug glycogen in the isopropanol precipitation). RNA purity was assessed via spectrometry (Nanodrop ND1000), and samples with 260/230 ratios lower than 1.7 were cleaned via a further isopropanol precipitation. RNA integrity was confirmed for a representative panel of samples via gel electrophoresis.

### cDNA synthesis

cDNA was prepared using the RTnanoscript2 kit (Primerdesign), using 1.6ug total RNA per reaction, with oligodT and random nonamer priming. cDNAs were subsequently diluted 1/20 in nuclease-free H2O to give a final cDNA concentration of ~4ng.ul-1 (assuming 1:1 conversion). Samples were stored at -20°C.

### qPCR

Reactions were carried out using PrecisionPLUS SYBR green mastermix (Primerdesign) in duplicate or triplicate, using 2ul diluted cDNA per well (approx. 8ng). Cycling used a CFX384 thermal cycler (BioRad) in three-step PCR (95°C, 15sec; 60°C, 20sec; 72°C, 20sec for 40 cycles) with subsequent melt curves performed for all reactions. Quantification cycle (Cq) values were determined by regression, and (where necessary) converted to relative quantities (RQ). Primers to *FBXO38*, *FBXW2*, *MON2*, *ZFP91*, *HTATSF1*, *GAPDH*, *ACTB*, *18S*, *CDC40*, *SDHA*, *RPL13a*, *CSNK2A2*, *AP3D1*, *PAK1IP1* and *B2M* were taken from the geNorm and geNormPLUS primer sets (Primerdesign) and sequences are thus proprietary. In accordance with the amended MIQE guidelines for proprietary primer sequences [54] we have included anchor nucleotide and context lengths for the amplicons used (see S2 Appendix). Primers to *HPRT1* were the well-validated pan-species set taken from [55], and have the following sequence:

HPSF F 5’-GGACTAATTATGGACAGGACTG-3’

HPSF R 5’-GCTCTTCAGTCTGATAAAATCTAC-3’

All primer sets produced single amplicons and all reactions were of comparable efficiency (95–105%). Additional detail regarding primer validation can be found in S2 Appendix.

### Analyses

geNorm, Bestkeeper and ungrouped Normfinder analyses were conducted on our entire dataset, and also on subsets of our data (number of samples indicated in brackets) as follows:

- Healthy samples only (N = 63)
- Dystrophic samples only (N = 63)
- 6 week samples only (N = 42)
  ○ 6 week healthy only (N = 21)
  ○ 6 week dystrophic only (N = 21)
- 10 week samples only (N = 42)
  ○ 10 week healthy only (N = 21)
  ○ 10 week dystrophic only (N = 21)
- 24 week samples only (N = 42)
  ○ 24 week healthy only (N = 21)
  ○ 24 week dystrophic only (N = 21)

Given the research interest in specific muscle groups, and the distinct metabolic and disease-specific responses of particular muscles to dystrophic progression, we also used several more focussed subsets. These subsets reduced our dataset to the level of individual muscles of particular research value (diaphragm, heart, TA), or excluded muscles with unique properties (i.e. a dataset containing non-respiratory skeletal muscles: all muscles except the fibrosis-prone slow, oxidative diaphragm and the non-skeletal heart muscle). We did not further divide these subsets by healthy/dystrophic, as such an approach would in most cases provide datasets of only nine samples, greatly increasing susceptibility to noise. The datasets were thus as follows:

- Non-respiratory skeletal muscle only (TA/GC/Q/Tri/BW: N = 90)
- Diaphragm only (N = 18)
- Heart only (N = 18)
- TA only (N = 18)

For grouped Normfinder analysis, grouping criteria were applied to the whole dataset (or, where applicable to allow at least two members per group, to subsets as described above). Grouping criteria used were as follows:

- Individual animal (18 groups)
- Healthy/dystrophic (2 groups)
- Muscle type (7 groups)
- Age (3 groups)

Bestkeeper analysis used raw Cq values, while both geNorm and Normfinder used linearised relative quantity (RQ) values.

### Statistics

Statistical analyses for category-specific expression differences used appropriate non-parametric tests. Healthy vs Dystrophic: Mann-Whitney U; Muscle-group, age, or muscle-group by healthy/dystrophic: one-way ANOVA with Sidak’s multiple comparisons test. Correlation coefficients (Pearson or Spearman’s rho) were determined as indicated. All statistical analysis was performed using GraphpadPRISM software or Microsoft excel.

## Results

### Cq distributions

Cq values for our panel of candidate genes (S1A Fig) covered a broad range of expression levels, from the very highly expressed (*18S*, *GAPDH*) to the more modestly expressed (*FBXW2*, *CSNK2A2*). Expression levels were also highly comparable with those reported for these genes in the Human BodyMap 2.0 data [56] (S1B Fig). Unexpectedly however, three of our candidate genes (*FBXO38*, *MON2*, *ZFP91*) showed very high variation in expression, and moreover failed to provide quantitative amplicons in several of our samples, with near limit-of-detection levels in others (in both cases, invariably those taken from 24 week old dystrophic mice). While of academic interest (and clearly a finding that highlights the stark differences between healthy and dystrophic muscle), missing data combined with innately high variability immediately rendered these genes unsuitable for use in our study. These genes were thus omitted from subsequent analysis. Distribution of within-gene Cq values for our remaining thirteen candidates were lowest in *AP3D1* and *CSNK2A2*, and highest in *FBXW2* (S1 Table).

### geNorm analysis

**Fig 2:**
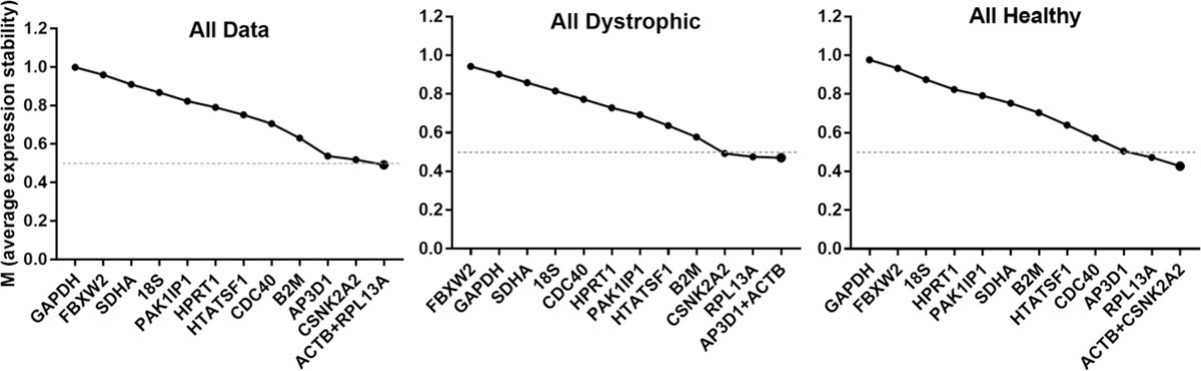
geNorm analysis. Representative outputs of the geNorm algorithm. geNorm ranking by average expression stability M (left to right: least stable to most stable) for the entire dataset, or dystrophic or healthy samples alone (as indicated). Dashed line: M = 0.5 (threshold of stability for strong candidates).

The pairwise approach of the geNorm algorithm [17] calculates a stability factor M for each gene (see S1 Appendix), a measure of the extent to which expression of that gene correlates with other genes in the dataset. Iterative removal of the gene exhibiting the least correlation (highest M), followed by recalculation of M values allows the dataset to be reduced until only a single pair of highly-correlated genes remain: the ‘best pair’. The M value of this final pair, and the values of each discarded gene allow candidate genes to be ranked from least to most stable (lowest M). By convention, candidates with M values < 0.5 are generally considered to be appropriate reference genes, though for samples expected to exhibit high transcriptional variability (such as comparison of multiple cell types, or samples derived from tumours) values < 1.0 are adjudged acceptable [57]. Analysis of our dataset as a whole, or assessed as healthy or dystrophic samples alone (Fig 2, Table 2), revealed that *ACTB*, *CSNK2A2*, *RPL13a* and *AP3D1* consistently ranked highly (indeed, *ACTB* represented one member of the best pair in all cases). Stability as a whole was however modest, as might be expected for such varied samples: for the analysis of our entire dataset, only the pairing of *ACTB* and *RPL13a* fell below the 0.5 threshold (though healthy/dystrophic only datasets showed marginally higher stability, with several genes achieving this benchmark). When analysed by sub-category (Table 2 and S2 Fig) a similar pattern was observed, with *ACTB*, *CSNK2A2*, *RPL13a* and *AP3D1* near-universally appearing within the top five highest scoring candidates, suggesting these genes are suitable both for broad panels of muscles and for individual muscle groups (such as diaphragm).

**Table 2:** geNorm rankings.

	All data	All healthy	All DMD	All 6wk	All 10wk	All 24wk	All skeletal muscle	All DIA	All Hearts	All TA	6wk healthy	6wk DMD	10wk healthy	10wk DMD	24wk healthy	24wk DMD
Best Pair	ACTB + RPL13A	ACTB + CSNK2A2	AP3D1 + ACTB	AP3D1 + CSNK2A2	AP3D1 + RPL13A	AP3D1 + ACTB	AP3D1 + CSNK2A2	ACTB + RPL13A	HTATSF1 + CSNK2A2	ACTB + RPL13A	ACTB + CSNK2A2	AP3D1 + CSNK2A2	AP3D1 + RPL13A	ACTB + RPL13A	AP3D1 + RPL13A	AP3D1 + ACTB
Most	CSNK2A2	**RPL13A**	**RPL13A**	RPL13A	**CSNK2A2**	CSNK2A2	**RPL13A**	AP3D1	**CDC40**	AP3D1	**RPL13A**	**HPRT1**	**CSNK2A2**	**AP3D1**	ACTB	HTATSF1
stable	AP3D1	AP3D1	CSNK2A2	ACTB	**ACTB**	RPL13A	ACTB	CSNK2A2	**AP3D1**	CSNK2A2	**AP3D1**	**RPL13A**	**HPRT1**	**CSNK2A2**	CSNK2A2	RPL13A
	B2M	CDC40	B2M	CDC40	B2M	HTATSF1	B2M	HTATSF1	**RPL13A**	HPRT1	CDC40	**18S**	**ACTB**	HPRT1	HTATSF1	CSNK2A2
	CDC40	HTATSF1	HTATSF1	HPRT1	CDC40	HPRT1	CDC40	PAK1IP1	**SDHA**	CDC40	HTATSF1	**CDC40**	**CDC40**	B2M	HPRT1	HPRT1
	*HTATSF1*	B2M	PAK1IP1	HTATSF1	18S	PAK1IP1	HPRT1	GAPDH	HPRT1	GAPDH	HPRT1	**ACTB**	SDHA	HTATSF1	CDC40	PAK1IP1
	*HPRT1*	*SDHA*	HPRT1	18S	*PAK1IP1*	SDHA	*HTATSF1*	*B2M*	ACTB	HTATSF1	PAK1IP1	**FBXW2**	B2M	PAK1IP1	*SDHA*	SDHA
	*PAK1IP1*	*PAK1IP1*	*CDC40*	FBXW2	*HTATSF1*	*B2M*	*PAK1IP1*	*SDHA*	GAPDH	SDHA	B2M	HTATSF1	HTATSF1	CDC40	*PAK1IP1*	B2M
	*18S*	*HPRT1*	*18S*	PAK1IP1	*HPRT1*	*CDC40*	*GAPDH*	*18S*	PAK1IP1	*B2M*	SDHA	B2M	PAK1IP1	18S	*B2M*	*18S*
	*SDHA*	*18S*	*SDHA*	*B2M*	*GAPDH*	*18S*	*18S*	*HPRT1*	18S	*18S*	*FBXW2*	PAK1IP1	18S	*GAPDH*	*18S*	*FBXW2*
Least	*FBXW2*	*FBXW2*	*GAPDH*	*SDHA*	*SDHA*	*FBXW2*	*FBXW2*	*CDC40*	*B2M*	*PAK1IP1*	*18S*	SDHA	*GAPDH*	*FBXW2*	*GAPDH*	*CDC40*
stable	*GAPDH*	*GAPDH*	*FBXW2*	*GAPDH*	*FBXW2*	*GAPDH*	*SDHA*	*FBXW2*	*FBXW2*	*FBXW2*	*GAPDH*	GAPDH	*FBXW2*	*SDHA*	*FBXW2*	*GAPDH*

geNorm results for the entire dataset or subsets (as indicated), ranked from highest scoring pair (top) to least stable candidate (bottom). Bold: score < 0.5 (threshold for suitability); italics: score > 0.75 (exceptionally poor candidates)

Interestingly, assessment of heart samples alone revealed that *HTATSF1* appears uniquely stable in this tissue, here forming one half of the best pair despite scoring comparatively poorly under all other conditions. Conversely, *ACTB* fared less well in this specific tissue. Heart samples also exhibited higher stability as a whole, possibly reflecting the relatively mild cardiac phenotype in *mdx* mice of the ages studied. In marked contrast, overall sub-category stability values for skeletal muscle clearly illustrate the transcriptional differences between dystrophic and healthy tissue: when categorised by age alone (thus comparing age-matched healthy and dystrophic tissue) even the best scoring candidates exhibited M values close to 0.5, but further sub-division into separate healthy/diseased categories enhanced stability values considerably.

Analysis also reveals that *FBXW2*, *SDHA*, *GAPDH* and *18S* are exceptionally poor candidates, with M values > 0.75 under essentially all dataset combinations.

geNorm analysis additionally indicates the change in pairwise variation elicited by increasing the number of reference genes beyond the best pair (S2 Table): while increasing from two to three (or four) lowered variation, the threshold for acceptable variation is <0.2, and the best pair was sufficient in all cases.

### Bestkeeper analysis

**Fig 3:**
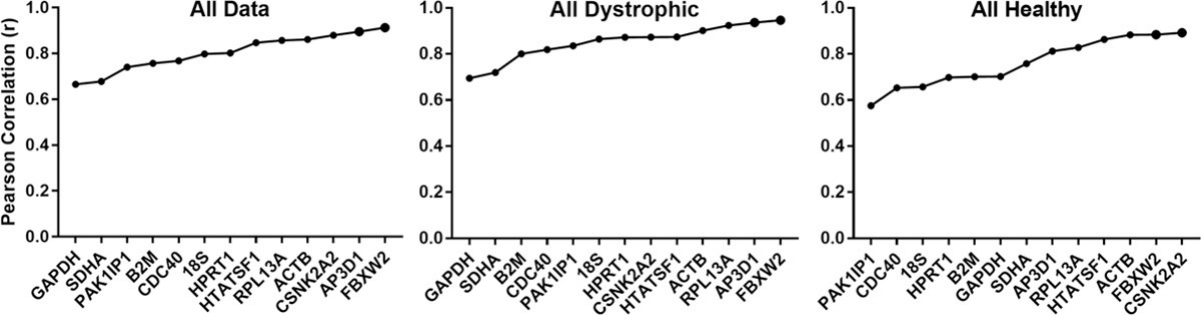
BestKeeper analysis. Representative outputs of the BestKeeper algorithm. Coefficient of correlation values for the reference gene candidates are shown for the entire dataset, or dystrophic or healthy samples alone (as indicated), ranked (left to right) from least stable to most stable.

This algorithm substitutes the iterative pairwise method of geNorm with a pairwise comparison of each gene individually against the geometric average of *all* genes, essentially determining which gene best reflects the behaviour of the dataset as a whole [18]. Genes can thus be ranked by their Pearson correlation coefficient (r), where r = 1 represents perfect correlation. As shown (Fig 3, Table 3 and S3 Fig), and in agreement with geNorm analysis, *ACTB*, *CSNK2A2*, *RPL13a* and *AP3D1* consistently score highly, while *SDHA*, *GAPDH* and *18S* tended to show lower correlation values. Similarly, *HTATSF1* was revealed to be particularly suited to normalization of heart expression data. In marked contrast to geNorm, however, *FBXW2* (the gene exhibiting the most variable expression in our dataset, and one of lowest scoring under geNorm analysis) here unexpectedly represented one of the highest scoring candidates, often with r > 0.9.

**Table 3:** BestKeeper rankings.

	All data	All healthy	All DMD	All 6wk	All 10wk	All 24wk	All skeletal muscle	All DIA	All Hearts	All TA	6wk healthy	6wk DMD	10wk healthy	10wk DMD	24wk healthy	24wk DMD
Most	**FBXW2**	**CSNK2A2**	**FBXW2**	**HPRT1**	**FBXW2**	**HTATSF1**	**FBXW2**	**RPL13A**	**HTATSF1**	**FBXW2**	**ACTB**	**CSNK2A2**	**CSNK2A2**	**AP3D1**	**HTATSF1**	**ACTB**
stable	**AP3D1**	**FBXW2**	**AP3D1**	**CSNK2A2**	HPRT1	**FBXW2**	**CSNK2A2**	**AP3D1**	**CSNK2A2**	18S	**HPRT1**	**AP3D1**	ACTB	**RPL13A**	**FBXW2**	**HTATSF1**
	**CSNK2A2**	**ACTB**	**RPL13A**	**RPL13A**	HTATSF1	**ACTB**	AP3D1	**HTATSF1**	**AP3D1**	HPRT1	**CDC40**	HPRT1	SDHA	**FBXW2**	**ACTB**	**FBXW2**
	**ACTB**	**HTATSF1**	**ACTB**	**ACTB**	RPL13A	**AP3D1**	CDC40	**FBXW2**	**CDC40**	AP3D1	**CSNK2A2**	FBXW2	RPL13A	**ACTB**	**AP3D1**	**RPL13A**
	**RPL13A**	RPL13A	**HTATSF1**	**FBXW2**	CSNK2A2	**CSNK2A2**	ACTB	**ACTB**	**FBXW2**	RPL13A	**HTATSF1**	HTATSF1	FBXW2	GAPDH	**CSNK2A2**	**AP3D1**
	HTATSF1	AP3D1	**CSNK2A2**	**CDC40**	AP3D1	RPL13A	RPL13A	**GAPDH**	**RPL13A**	CDC40	**RPL13A**	CDC40	HPRT1	HPRT1	**18S**	**HPRT1**
	HPRT1	SDHA	**HPRT1**	**AP3D1**	GAPDH	18S	HPRT1	**B2M**	**HPRT1**	GAPDH	FBXW2	RPL13A	B2M	CSNK2A2	**GAPDH**	CSNK2A2
	18S	GAPDH	**18S**	**HTATSF1**	SDHA	SDHA	18S	**CSNK2A2**	**SDHA**	ACTB	AP3D1	*SDHA*	AP3D1	HTATSF1	RPL13A	PAK1IP1
	CDC40	B2M	PAK1IP1	**18S**	PAK1IP1	B2M	HTATSF1	PAK1IP1	**GAPDH**	CSNK2A2	SDHA	*18S*	HTATSF1	B2M	SDHA	B2M
	B2M	HPRT1	CDC40	B2M	*B2M*	PAK1IP1	PAK1IP1	18S	**ACTB**	SDHA	B2M	*GAPDH*	*GAPDH*	18S	B2M	SDHA
	PAK1IP1	18S	B2M	PAK1IP1	*ACTB*	GAPDH	B2M	SDHA	**18S**	HTATSF1	18S	*B2M*	*CDC40*	*PAK1IP1*	PAK1IP1	18S
Least	SDHA	CDC40	SDHA	*SDHA*	*CDC40*	HPRT1	GAPDH	HPRT1	PAK1IP1	*B2M*	*PAK1IP1*	*ACTB*	*PAK1IP1*	*CDC40*	*HPRT1*	CDC40
stable	GAPDH	*PAK1IP1*	GAPDH	*GAPDH*	*18S*	CDC40	*SDHA*	*CDC40*	B2M	*PAK1IP1*	*GAPDH*	*PAK1IP1*	*18S*	*SDHA*	*CDC40*	GAPDH

Bestkeeper results for the entire dataset or subsets (as indicated), ranked from top to bottom by Pearson correlation (r) with the BestKeeper. Bold: r > = 0.85; italics: r < 0.6

### Normfinder

**Fig 4:**
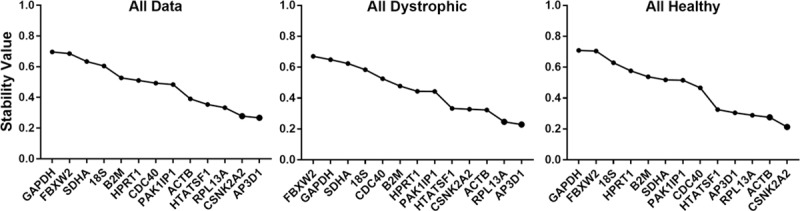
Normfinder analysis (ungrouped). Representative outputs of the Normfinder algorithm. Stability values (left to right: least stable to most stable) for the reference gene candidates are shown for the entire dataset or dystrophic or healthy samples alone (as indicated).

Unlike the pairwise approaches of other two algorithms, the Normfinder algorithm assesses absolute expression stability, either within the dataset as a whole, or within and between subgroups specified by the user [19]. Each gene is thus effectively evaluated individually, rather than by comparison with other candidates: Normfinder can identify single highly-stable gene candidates that geNorm and Bestkeeper may not. As shown (Fig 4, Table 4 and S4 Fig) even under this alternative assessment methodology, *CSNK2A2*, *RPL13a* and *AP3D1* again were consistently ranked highly, with *ACTB* also rated as highly stable under most dataset combinations. *HTATSF1* again scored highly in heart samples alone, though here this gene was also ranked comparatively highly overall (especially in 24 week-old samples).

**Table 4:** Normfinder rankings (ungrouped).

	All data	All healthy	All DMD	All 6wk	All 10wk	All 24wk	All skeletal muscle	All DIA	All Hearts	All TA	6wk healthy	6wk DMD	10wk healthy	10wk DMD	24wk healthy	24wk DMD
Most	**AP3D1**	**CSNK2A2**	**AP3D1**	**CSNK2A2**	**AP3D1**	**HTATSF1**	**CSNK2A2**	**AP3D1**	**CSNK2A2**	**AP3D1**	**ACTB**	**CSNK2A2**	**CSNK2A2**	**AP3D1**	**HTATSF1**	**HTATSF1**
stable	**CSNK2A2**	**ACTB**	**RPL13A**	**AP3D1**	**CSNK2A2**	**AP3D1**	**AP3D1**	**RPL13A**	**HTATSF1**	**CDC40**	**CSNK2A2**	**AP3D1**	**AP3D1**	**RPL13A**	**AP3D1**	**ACTB**
	**RPL13A**	**RPL13A**	**ACTB**	**CDC40**	**RPL13A**	**ACTB**	**CDC40**	**HTATSF1**	**RPL13A**	**HPRT1**	**HTATSF1**	**18S**	**RPL13A**	**ACTB**	**ACTB**	**RPL13A**
	**HTATSF1**	**AP3D1**	**CSNK2A2**	**HTATSF1**	CDC40	**CSNK2A2**	**RPL13A**	**ACTB**	**CDC40**	**RPL13A**	**RPL13A**	**HPRT1**	**ACTB**	**CSNK2A2**	**CSNK2A2**	**AP3D1**
	**ACTB**	**HTATSF1**	**HTATSF1**	**RPL13A**	HTATSF1	**RPL13A**	**HTATSF1**	CSNK2A2	**AP3D1**	**CSNK2A2**	**AP3D1**	**RPL13A**	**SDHA**	**HPRT1**	**RPL13A**	**CSNK2A2**
	PAK1IP1	CDC40	PAK1IP1	**ACTB**	HPRT1	PAK1IP1	ACTB	PAK1IP1	**SDHA**	ACTB	**CDC40**	**CDC40**	**HPRT1**	**HTATSF1**	18S	**HPRT1**
	CDC40	PAK1IP1	HPRT1	HPRT1	PAK1IP1	SDHA	PAK1IP1	GAPDH	**ACTB**	GAPDH	**HPRT1**	**HTATSF1**	**CDC40**	PAK1IP1	SDHA	PAK1IP1
	HPRT1	SDHA	B2M	18S	B2M	HPRT1	HPRT1	B2M	**HPRT1**	HTATSF1	PAK1IP1	**FBXW2**	HTATSF1	B2M	PAK1IP1	B2M
	B2M	B2M	CDC40	PAK1IP1	ACTB	B2M	GAPDH	SDHA	GAPDH	SDHA	FBXW2	**ACTB**	PAK1IP1	18S	B2M	SDHA
	*18S*	HPRT1	18S	B2M	18S	*18S*	B2M	18S	PAK1IP1	18S	B2M	B2M	B2M	CDC40	CDC40	*FBXW2*
	*SDHA*	*18S*	*SDHA*	FBXW2	*GAPDH*	*CDC40*	FBXW2	*HPRT1*	18S	B2M	SDHA	PAK1IP1	18S	GAPDH	*HPRT1*	*18S*
Least	*FBXW2*	*FBXW2*	*GAPDH*	*SDHA*	*SDHA*	*FBXW2*	*18S*	*CDC40*	*B2M*	*PAK1IP1*	*18S*	GAPDH	*GAPDH*	*FBXW2*	*GAPDH*	*CDC40*
stable	*GAPDH*	*GAPDH*	*FBXW2*	*GAPDH*	*FBXW2*	*GAPDH*	*SDHA*	*FBXW2*	*FBXW2*	*FBXW2*	*GAPDH*	SDHA	*FBXW2*	*SDHA*	*FBXW2*	*GAPDH*

Normfinder results for the entire ungrouped dataset or subsets (as indicated), ranked (top to bottom) from highest scoring (lowest stability value) to lowest scoring. Bold: stability <0.4; italics: stability > 0.6

In agreement with both other algorithms, *SDHA*, *18S* and *GAPDH* were assigned poor stability values, and in support of geNorm (but in dramatic contrast with BestKeeper), *FBXW2* was again consistently rated as one of the least stable genes in our dataset. Under grouped analysis (Table 5 and S3, S4 and S5 Tables) a similar pattern was observed, though this methodology also highlights categorical subtleties, permitting further inferences to be drawn. For example, while *CNSK2A2* and *AP3D1* appeared highly stable essentially regardless of grouping criterion, *ACTB* and (to a less dramatic extent) *RPL13a* tended to score highly when samples were grouped by age, muscle type or individual, but performed less well when grouped by healthy/dystrophic, perhaps suggesting these genes are not as well-suited to disease-specific comparisons as *CSNK2A2* and *AP3D1*. As with ungrouped analysis, *FBXW2*, *SDHA*, *GAPDH* and *18S* were rated as low stability candidates near-universally, with *SDHA* and *GAPDH* being particularly sensitive to grouping by muscle type.

**Table 5:** Normfinder rankings (grouped).

	All Samples				All Healthy			All Dystrophic		
	Animal	Disease	Muscle	Age	Animal	Muscle	Age	Animal	Muscle	Age
Best pair	**AP3D1** **+** **HTATSF1**	**HTATSF1** **+** **B2M**	**AP3D1** **+** **HTATSF1**	**AP3D1** **+** **CSNK2A2**	**HTATSF1** **+** **CSNK2A2**	**AP3D1** **+** **HTATSF1**	**AP3D1** **+** **HTATSF1**	**PAK1IP1** **+** **RPL13A**	**AP3D1** **+** **CSNK2A2**	**AP3D1** **+** **PAK1IP1**
Most	**CSNK2A2**	**AP3D1**	**CSNK2A2**	**ACTB**	**CSNK2A2**	**CSNK2A2**	**RPL13A**	**RPL13A**	**AP3D1**	**RPL13A**
stable	**AP3D1**	**HPRT1**	**AP3D1**	**AP3D1**	**RPL13A**	**ACTB**	**CSNK2A2**	**AP3D1**	**CSNK2A2**	**AP3D1**
	HTATSF1	**CDC40**	**ACTB**	**CSNK2A2**	**ACTB**	**AP3D1**	**B2M**	**CSNK2A2**	**ACTB**	**CDC40**
	RPL13A	**CSNK2A2**	**B2M**	**PAK1IP1**	**HTATSF1**	HTATSF1	**ACTB**	ACTB	**RPL13A**	**ACTB**
	CDC40	**18S**	**HTATSF1**	**RPL13A**	**AP3D1**	RPL13A	**AP3D1**	HTATSF1	**HTATSF1**	**HTATSF1**
	PAK1IP1	**FBXW2**	**RPL13A**	**SDHA**	B2M	PAK1IP1	**HTATSF1**	PAK1IP1	**HPRT1**	**CSNK2A2**
	ACTB	**PAK1IP1**	**PAK1IP1**	**HTATSF1**	CDC40	CDC40	**GAPDH**	CDC40	PAK1IP1	**PAK1IP1**
	B2M	**HTATSF1**	18S	**B2M**	SDHA	B2M	**PAK1IP1**	B2M	B2M	SDHA
	HPRT1	**B2M**	HPRT1	**CDC40**	PAK1IP1	18S	**FBXW2**	HPRT1	18S	B2M
	GAPDH	**RPL13A**	FBXW2	**GAPDH**	GAPDH	HPRT1	CDC40	GAPDH	FBXW2	GAPDH
	*FBXW2*	**GAPDH**	CDC40	**FBXW2**	FBXW2	SDHA	SDHA	SDHA	CDC40	HPRT1
Least	*SDHA*	ACTB	SDHA	**HPRT1**	18S	*FBXW2*	18S	*18S*	SDHA	18S
stable	*18S*	SDHA	*GAPDH*	18S	HPRT1	*GAPDH*	HPRT1	*FBXW2*	*GAPDH*	FBXW2

Normfinder results for the entire dataset or healthy/diseased subsets grouped by different criteria (as indicated: top row; datasets, second row; criterion), ranked from highest scoring (lowest stability value) to lowest scoring (see supplementary data for extended grouped analysis). Grouped analysis also suggests the best pair of genes for normalization (third row), (not necessarily the highest scoring individually). Bold: stability <0.25; italics: stability > 0.4

Grouped analysis further suggests a ‘best pair’: two genes that may be of low individual stability, but in opposing senses, such that when combined they outperform any single candidate within the dataset analysed. Of the 41 dataset groupings analysed (Table 5 and S6 Table), *AP3D1* formed one part of the best pair in 30 cases, while *CSNK2A2* appeared 17 times. Interestingly, *ACTB* and *RPL13a* were rarely nominated (2 and 3 times respectively) while *HTATSF1* earned 21 nominations.

Taking all three algorithm outputs together, our data suggests that *CSNK2A2*, *AP3D1*, *RPL13a* and *ACTB* are suitable for normalizing gene expression in healthy and dystrophic mice regardless of animal age or muscle studied. *RPL13a* and *ACTB* may be less optimal for specific healthy/dystrophic comparisons if used individually, however as shown by geNorm these genes remain very strong if used as part of a set.

### Validation of gene candidates

**Fig 5:**
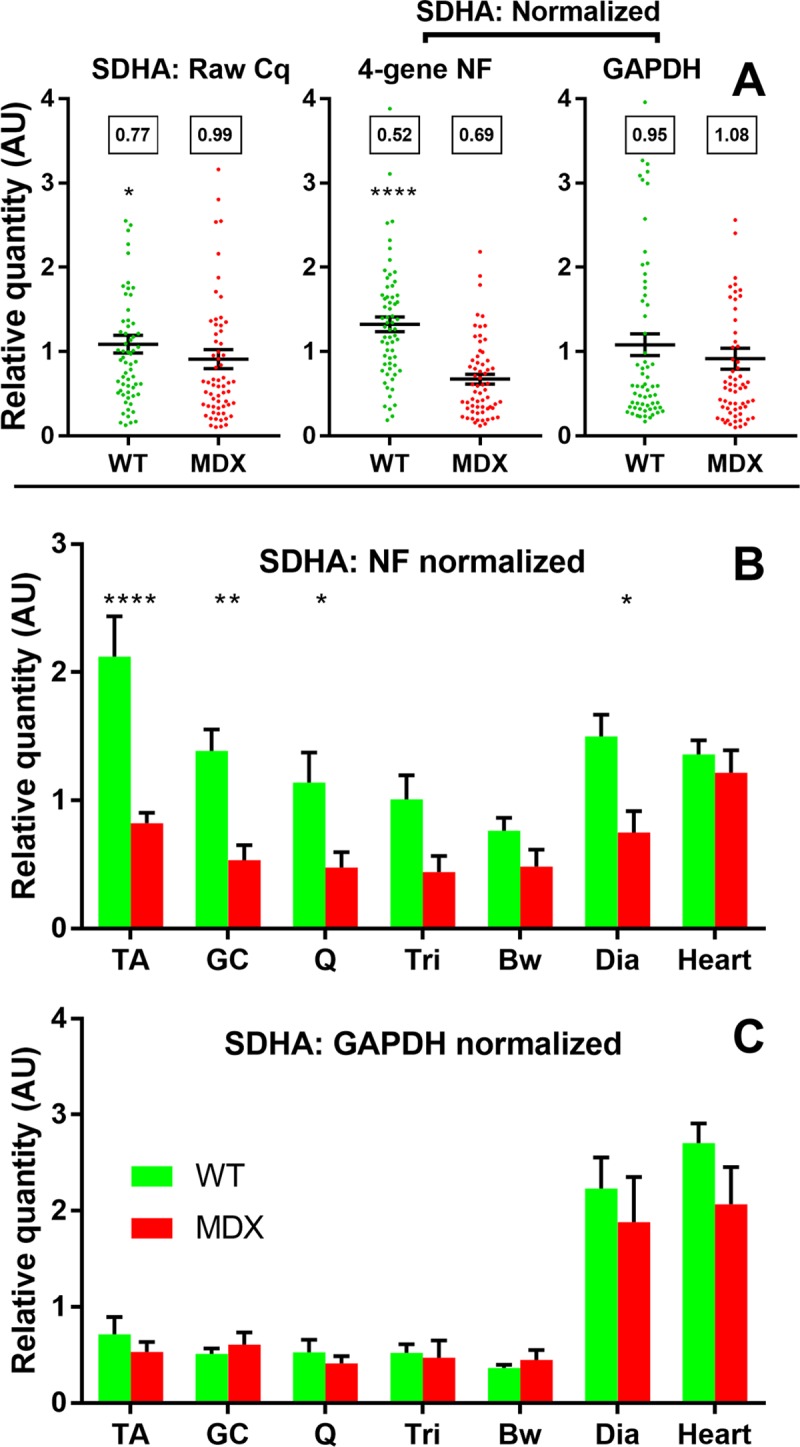
Succinate dehydrogenase subunit A expression. **(A)** Expression data for *SDHA* in all healthy and dystrophic samples, either raw relative quantity (RQ) values (left); normalized to the geometric mean of *ACTB*, *RPL13a*, *CSNK2A2* and *AP3D1* (centre); or to *GAPDH* (right). **(B)** and **(C)** expression data for SDHA in healthy and dystrophic samples sorted by muscle group (as indicated) normalized to the geometric mean of *ACTB*, *RPL13a*, *CSNK2A2* and *AP3D1* **(B)** or to *GAPDH* **(C)**. Data shown either as individual sample RQ values (●) and means +/- SEM, or means + SEM alone. *:P<0.05; **:P<0.01,****:P<0.0001 (Mann-Whitney U test (healthy/dystrophic) or one-way ANOVA with Sidak’s multiple comparisons test (healthy/dystrophic by muscle)). Boxes: Coefficient of Variation.

To assess the validity of the four high-scoring gene candidates (*CSNK2A2*, *AP3D1*, *RPL13a* and *ACTB*), we employed a within-dataset strategy we have used previously: using a normalization factor generated from our highest-scoring genes (NF: geometric mean of the four candidates above) to normalize low-scoring genes from our panel. In addition, as *GAPDH* is often employed as a reference in the literature, this gene (here scoring poorly) was also used to normalize other low-scoring genes for comparative purposes. We first addressed *SDHA*: in canine muscle this gene is a high-scoring and universally-suitable candidate [20]; conversely, our work here suggests this gene is highly unstable in mouse muscle under all algorithms and essentially all dataset comparisons. Normalization of *SDHA* expression via our NF data reveals this gene to not only exhibit a highly muscle-specific transcriptional program, but also to be markedly reduced in dystrophic tissue (with the exception of the mildly-affected heart muscle) (Fig 5A & 5B). In contrast, following normalization with *GAPDH* alone this muscle-specificity appears grossly simplified, and disease-associated changes are no longer apparent at individual muscle or even entire dataset level (Fig 5A & 5C). The coefficient of variation (CoV, a measure of the spread of data) is also substantially reduced by normalization with our four-gene NF, but increases following normalization with *GAPDH*.

**Fig 6:**
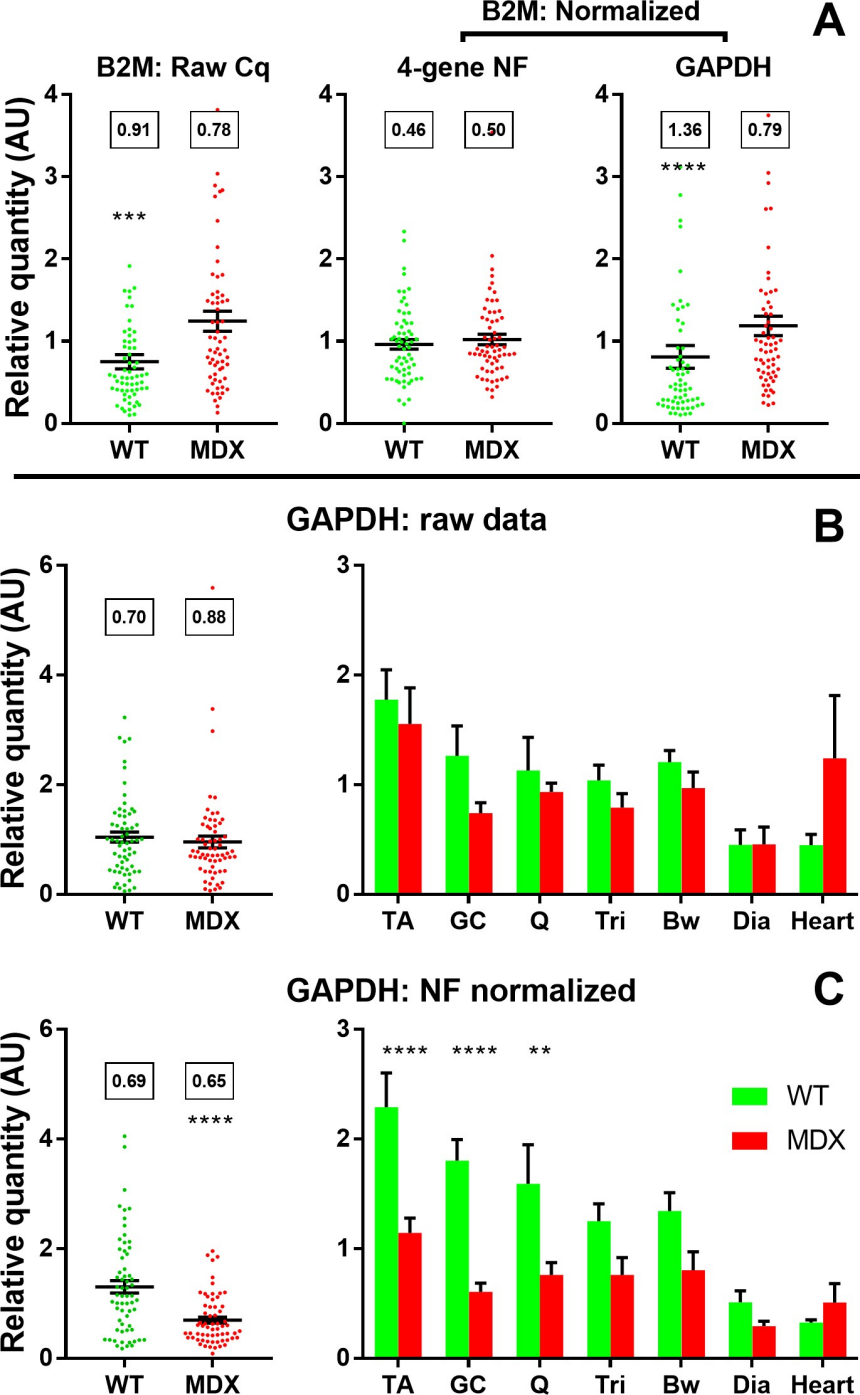
Beta 2 microglobulin and Glyceraldehyde 3-phosphate dehydrogenase expression. **(A)** Expression data for *B2M* in all healthy and dystrophic samples, either raw relative quantity (RQ) values (left); normalized to the geometric mean of *ACTB*, *RPL13a*, *CSNK2A2* and *AP3D1* (centre); or to *GAPDH* (right). **(B)** and **(C)** Expression data for *GAPDH* in all healthy and dystrophic samples (left), or healthy and dystrophic samples sorted by muscle group (as indicated, right), either raw **(B)** or normalized to the geometric mean of *ACTB*, *RPL13a*, *CSNK2A2* and *AP3D1* **(C)**. All data shown either as means +/- SEM plus individual sample RQ values (●) or as means + SEM alone. **:P<0.01,***:P<0.001,****:P<0.0001 (Mann-Whitney U test (healthy/dystrophic) or one-way ANOVA with Sidak’s multiple comparisons test (healthy/dystrophic by muscle)). Boxes: Coefficient of Variation.

Our second candidate was *B2M*: this gene (beta-2-microglobulin) is actively disease-associated in dogs [20], showing an up-regulation of approximately 2-fold in dystrophic canine muscle (likely reflecting elevated numbers of immune cells in damaged tissue). Though this gene has been used in the literature [39], performance of this gene in our mouse panel was mediocre, and as shown in Fig 6A, the raw (non-normalized) data for *B2M* in our mouse muscle dataset does appear to suggest a highly significant (P = 0.0002) increase in *B2M* expression in dystrophic tissue. Crucially, however: after normalization to the geometric mean of *CSNK2A2*, *AP3D1*, *RPL13a* and *ACTB* this difference disappears entirely (P = 0.72). This unexpected finding appears to be genuine: as shown, the coefficients of variation are markedly reduced by normalization, exactly as would be expected if such normalization is effective. Conversely, normalization to *GAPDH* exaggerates this apparently aberrant difference (P<0.0001), and again also increases CoV values.

Given these findings, it seemed prudent to use our NF to normalize *GAPDH* data: as shown (Fig 6B), raw *GAPDH* data shows no overt disease-associated expression pattern, though does display some muscle-group specificity. Following normalization (Fig 6C), *GAPDH* exhibits marked muscle-specific variation in expression, and is further revealed to be sharply downregulated (~2-fold) in all dystrophic skeletal muscle (but not heart).

**Fig 7:**
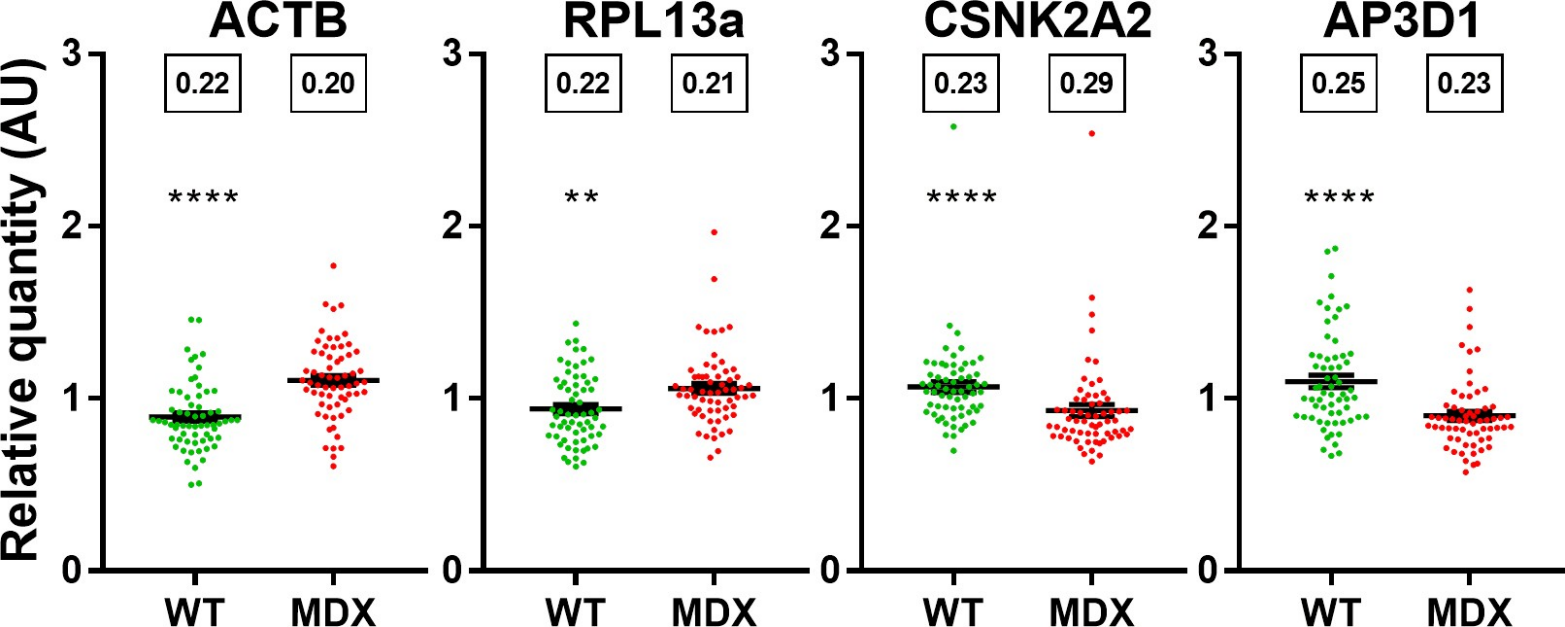
Expression of the four high scoring genes. Expression data for *ACTB*, *RPL13a*, *CSNK2A2* and *AP3D1* (as indicated) in all healthy and dystrophic samples, individually normalized to the geometric mean of *ACTB*, *RPL13a*, *CSNK2A2* and *AP3D1*. Data shown as individual sample relative quantity values (●) and means +/- SEM. **:P<0.01,****:P<0.0001 (Mann-Whitney U test). Boxes: Coefficient of Variation.

*GAPDH* is a widely-used reference gene in the *mdx* mouse, and one could rightly posit that these disease-associated decreases in expression might instead be an artefact generated by a poorly-chosen normalization factor composed of genes that all show increased expression in diseased tissue. We thus subjected each of our high-scoring genes to normalization with our NF (a slightly unorthodox approach that means each gene is effectively normalized–in part- to itself, but which allows each gene’s individual bias with respect to the NF to be assessed, and moreover allows all four normalized genes to be directly compared). Raw data suggests a dystrophy-associated increase in all cases (S5A Fig) however following normalization (Fig 7 and S5B Fig) all four genes show marked reduction in CoV, and none of the genes exhibit any significant muscle specificity. Nevertheless, our data suggests each gene is indeed disease-associated to a biologically mild (~20% change) but statistically highly-valid (P = 0.002 for *RPL13a*, P<0.0001 otherwise) extent. Crucially, however, the genes differ in the direction of change: *ACTB* and *RPL13a* both show a ~20% increase in dystrophic tissue, while *CSNK2A2* and *AP3D1* both show a ~20% decrease in dystrophic tissue. Our data thus suggest that (much as for selection of the best pair in grouped Normfinder analysis) while no single gene is wholly stable between healthy and dystrophic muscle, a highly stable normalization factor can be generated by geometric averaging of two (or four) genes that exhibit small disease-specific changes in equal but opposite directions.

## Discussion

A panel of reference genes suitable for normalizing gene expression in both healthy and dystrophic tissue (regardless of age or muscle group) would represent a valuable tool in the DMD research tool-kit, ostensibly permitting otherwise quite disparate samples (such as aged diaphragm and young *tibialis anterior*) to be empirically compared and evaluated, both within and between research groups. Dystrophic muscle is however host to a complex mixture of cell types, and moreover a mixture that changes with age, frequency of use and muscle type: it is by no means guaranteed that any set of genes might be appropriate for normalizing expression between dystrophic muscles, let alone between such muscles and matched healthy tissue. The study presented here attempts to address this question in a rigorous and comprehensive fashion, with a dataset using 13 genes and fully 126 samples, allowing assessment of gene suitability in multiple muscles, including the TA, diaphragm and heart, taken from *mdx* and strain-matched healthy mice at three disease- and research-relevant ages (6 week early disease, 10 week disease progression and 24 week established disease, with all three being commonly employed time-points for therapeutic trials). As shown, our data reveal that *CSNK2A2*, *AP3D1*, *RPL13a* and *ACTB* appear to represent a universal mouse panel (albeit not without caveats). These four genes score highly under geNorm, Normfinder and BestKeeper analysis, both within the dataset as a whole and when assessed as subsets of the data, suggesting a high level of stability. Further investigation clarified this stability: all four genes exhibit no age or muscle-group specific changes, however all four genes do show moderate (but statistically-significant) disease-associated changes (Fig 7). The changes are small and indeed below the threshold of significance when considered on an individual muscle level: only with the greater statistical power afforded by more systematic analysis do these changes reveal themselves (illustrating a major strength of our large and comprehensive dataset). Such a result might be considered problematic: use of any one of these genes as a reference would introduce a small (~20%) but nevertheless consistent disease-specific bias. The significance of our findings is that this bias can be effectively (and easily) eliminated, as the differences exhibited are almost perfect mirror images of each other: the extent to which *ACTB* and *RPL13a* are increased in dystrophic tissue is near-exactly matched by the extent to which *CSNK2A2* and *AP3D1* are decreased. A normalization factor prepared by geometric average of all four (or indeed one of each sign) should exhibit no net disease-associated behaviour, and would thus be entirely appropriate for normalizing gene expression data taken from both healthy and dystrophic tissue, regardless of animal age or muscle type studied.

For studies concerned exclusively with heart tissue, our data further reveal that while these four genes also tend to score highly in this tissue, the gene *HTATSF1* exhibits marked stability specifically in cardiac samples (outperforming many of the other four genes under all three algorithms). Of the muscles studied here, the heart is certainly the most unique, thus it is not surprising (and indeed, arguably encouraging) that such tissue-specific differences exist.

*CSNK2A2* and *AP3D1* both score highly in murine cell culture models of myogenesis (healthy or dystrophic) [34], remaining stable from proliferating myoblasts all the way to contractile myotubes. Given the relatively mild pathology of the *mdx* mouse (with minimal inflammation or fibrosis), the bulk of any given skeletal muscle is likely either mature muscle or muscle at various stages of regeneration: genes that remain stable through this process might well be expected to rank highly. As noted, both genes nevertheless exhibit a mild but consistent reduction in dystrophic muscle (perhaps reflecting the greater percentages of non-muscle cells within these tissues) but encouragingly these genes remain strong candidates regardless of whether 6, 10 or 24 week old samples are considered in isolation: as shown in Fig 1, each stage exhibits distinct pathological characteristics that correspond to marked changes in cellular composition. These two genes also score highly in the diaphragm, suggesting that the progressive accumulation of fibrotic scarring does not affect their suitability as references (at least up to 24 weeks of age). The performance of these genes in dogs is unknown, however we note these genes are absent from the *Canis familiaris* geNorm primer set (suggesting their stability may be mediocre).

The strong performance of *ACTB* is a surprise: we have previously shown this gene to be an exceptionally poor candidate in both myogenic cell cultures and healthy/dystrophic dogs [20, 34], and the expectation was that this gene would similarly fare poorly here. It is not immediately clear why this gene should be such a strong candidate in mouse muscle: expression of this cytoskeletal beta isoform falls during myogenesis (presumably as the increase in filamentous alpha-actin and other contractile proteins presses cytoskeletal requirements to the periphery) thus one would expect levels in healthy muscle to be low, while levels in dystrophic muscle -with its diverse cellular composition- to be elevated. As shown, expression of beta actin is indeed elevated in diseased muscle, and consistently so, but to only a comparatively minor extent.

*RPL13a* (which codes for a protein component of the large ribosomal subunit) is similarly identified as highly stable, but as with *ACTB*, also shows a small, but significant and consistent increase in expression within dystrophic muscle. A parsimonious interpretation of this increase might be that, courtesy of infiltrating inflammatory cells and proliferating myoblasts, dystrophic tissue simply contains a greater number of cells per unit mass. We note that *RPL13a* also scored highly in our canine panel, and indeed a number of studies have suggested that genes directly associated with the translational machinery tend to score highly as candidate reference genes [23, 28, 31, 32] under a variety of conditions, implying that translational components are highly stable. Such findings suggest that translation may not be limiting under most conditions, with even dramatic changes in cellular translational demand being readily accommodated by existing ribosomal content. A puzzling corollary, therefore, is the observation that *18S* ribosomal RNA performs so poorly under similar comparisons (both in dogs and mice). As the core RNA components of the ribosome, rRNAs must achieve stoichiometry with ribosomal proteins, and do so without the advantage of the amplification step inherent to translation (one mRNA -> many proteins): ribosomal RNA is thus incredibly abundant (as shown by low Cq values), and such abundance may explain this discrepancy. rRNAs are estimated to make up ~80% of total RNA, with tRNAs contributing a further 15% [58], thus measured RNA concentrations are primarily a reflection of ribosomal RNA concentration: small changes in rRNA fraction of a sample (say, 85% to 82%) would likely fall within the range of tolerable biological noise and thus have little effect on the total translational capacity of a cell, or the measured RNA concentration. The concomitant mRNA content of the two samples would however vary by 60% (an increase from 5% to 8% of total RNA), rendering *18S* a misleading metric of mRNA content. Use of extremely abundant RNAs as references already carries caveats with respect to efficiency and accuracy of measurement, thus it would seem ribosomal RNAs, despite the advantages in signal robustness their abundance ensures (and their widespread historical use), are poor references for study of gene expression.

In addition to *18S* as discussed above, our data also suggest that *GAPDH* -another frequently-employed reference gene- here performs remarkably poorly, as does *SDHA* (which conversely scored very highly in a canine model of DMD): indeed, both *GAPDH* and *SDHA* display prominent muscle-specific expression patterns, and both also show marked disease-specific changes. The fact that *SDHA* (succinate dehydrogenase subunit A) does not exhibit the same universal suitability as shown previously in dogs most likely stems from the inherent differences in skeletal muscle mitochondria between small and large mammals. In the muscle of dogs (and similarly, humans), fast glycolytic 2B fibres are essentially absent: even the fastest fibres present (2X) retain significant oxidative capacity, and the muscles of larger mammals consequently exhibit more metabolic homogeneity. In contrast, while all fibres of the mouse display proportionately higher mitochondrial volumes than larger mammal counterparts, they also exhibit marked metabolic compartmentalization: the fast, glycolytic muscles of mice are very fast, and very glycolytic (and vice versa for the slow, oxidative muscles) [59]. This compartmentalization is clearly reflected in the (normalized) expression of the mitochondrial marker *SDHA*, even between the comparatively fast skeletal muscles chosen for this study. *SDHA* also exhibits a prominent disease-specific reduction in muscle expression: this decrease may reflect elevated levels of non-muscle cells, or the large numbers of fast-twitch glycolytic regenerating fibres within active skeletal muscle, an explanation that would also account for the absence of such a decrease in the heart (as this tissue has limited regenerative capacity). Taken together, these findings render *SDHA* manifestly unsuitable as a reference in mouse muscle, instead serving here as a precautionary tale against extrapolating between model organisms.

Our finding that *GAPDH* also shows muscle-specific expression is not wholly surprising: several multiple-tissue transcriptomics studies have reported markedly higher expression of this gene in skeletal muscle than in heart [56, 60, 61]. *GAPDH* also exhibits a two-fold decrease in dystrophic tissue, however: a discovery that has potentially serious implications for studies using this gene as a reference for comparisons between healthy and diseased animals (or indeed treated vs untreated dystrophic tissues).

A final gene that performed exceptionally poorly was the Fbox/WD40 protein *FBXW2*, ranked as last or second-last by geNorm and Normfinder under most datasets, with the dramatic exception of BestKeeper analysis, where it was near-unanimously ranked highest. This was wholly unexpected: raw *FBXW2* Cq values show only moderate correlation with other candidate genes (S7 Table), and indeed exhibit the greatest sample-to-sample variation within our dataset; even before analysis the expectation was that this gene would fare poorly. One possible explanation for this discrepancy was that such high variability allowed this gene to exert a potent influence over the average generated by the BestKeeper algorithm, artificially enhancing its own correlation with that average. However, after removal of *FBXW2* from our dataset, the resultant (*FBXW2*-deleted) BestKeeper still correlates highly with *FBXW2* (Pearson correlation of 0.91 drops to 0.89, S6 Fig). We note however that this method ranks only by correlation with the BestKeeper, the average expression of all genes: comparisons between individual genes play no role in the final ranking. There is thus no *a priori* reason (beyond improbability) that a given gene might correlate only modestly with other individual candidates while still serendipitously correlating spectacularly well with the average of those candidates. Regardless of the underlying mechanism, such a finding illustrates the advantages of our approach: use of three different algorithms allows unexpected occurrences exactly like this to be readily identified and rightly dismissed as aberrant. We do not consider *FBXW2* to be a suitable reference gene here.

Taken as a whole, the above results are encouraging: our dataset is substantial, and we have endeavoured to cover a wide range of potential comparative scenarios so as to truly support our conclusions of universal suitability. We must nevertheless accept certain limitations: only three time-points were chosen, and while these were chosen to maximise utility to the *mdx* research field, our data is ostensibly only applicable to mice up to 24 weeks of age. In a similar vein, only 3 healthy and 3 dystrophic mice were taken for each time-point, limiting the levels of subdivision we could realistically achieve: while this is unlikely to affect the robustness of our dataset as a whole or under the subsets presented here, comparison (for example) of ‘dystrophic TA muscles alone’ would be a dataset of 9 samples, while ‘6 week dystrophic TA muscles alone’ would consist of 3, neither dataset large enough to confer confidence in the conclusions obtained. Five or six mice of each genotype might plausibly permit such analyses, but the numbers (210 or 252 samples respectively) render such studies impractical in terms of resources, time and sample-handling practicality. We also limited our investigations to only seven muscles (the TA, gastrocnemius, quadriceps, triceps, body wall, diaphragm and heart). Inclusion of the very fast EDL and slow, postural soleus muscle would be a welcome addition, but again for reasons of time and practicality (these muscles are also very small, limiting available tissue for RNA extraction) these were omitted. As a final caveat, this work addresses the *mdx* mouse (and non-dystrophic C57/Bl10 mice) alone: our final high-scoring candidate genes are well-supported, but we cannot claim these findings necessarily extend to other dystrophic mice such as the *mdx*^CV^ lines [62, 63] or the humanised *mdx Cmah*^-/-^ mouse [64].

These limitations conceded, we feel our data remains robust, comprehensive and compelling. Our nominated genes are not individually stable between healthy and dystrophic tissue (it may be the case that no such stable genes exist for comparisons in mouse), but nevertheless combine to produce a normalization factor that is stable regardless of disease, age, or muscle group. It would however be remiss not to address the pertinent observation that the contrasting behaviour of our high scoring genes (*CSNK2A2*, *AP3D1*, *RPL13a* and *ACTB*) and *GAPDH* could well be viewed from the opposite perspective. *GAPDH* (following normalization with our high-scoring candidates) is revealed to show marked muscle and disease-specific changes in expression, thus by extension our high-scoring candidates (following normalization with *GAPDH*) would show similar muscle and disease-specific changes in expression, but simply of the opposite sign. Both *ACTB* and *GAPDH* have been widely used as references in the *mdx* mouse (though almost never together), thus our findings may engender controversy regardless of interpretation. We have previously shown *GAPDH* to be a poor candidate both in canine muscle and murine cell cultures (healthy and dystrophic), though we note the very same analyses revealed *ACTB* to be a similarly poor choice. In the *mdx* mouse as shown here, *GAPDH* remains poor while *ACTB* appears to be a very strong candidate. Given the further observation that *B2M* is strongly disease-associated in a dog model of DMD, and appears similarly disease-associated in mouse if *GAPDH* is used as a reference but crucially not if our normalization factor (including *ACTB*) is used, our conclusions here warrant additional attention. The three algorithms employed here are unbiased, and the scoring obtained is an empirical measure of gene suitability under the constraints stipulated by the method in question, but each method has limitations. geNorm will select unstable but pairwise-matched candidates over highly stable candidates without an appropriate matching partner, while Normfinder will do the reverse; as illustrated by *FBXW2* here, BestKeeper may even produce aberrant results purely through chance. The fact that our four high-scoring candidates perform well under all three methods, both by pairwise comparisons (geNorm and BestKeeper) and by individual measure of stability (Normfinder: both within and between a wide range of possible sub-groupings) is however strong support, as is the concomitant observation that *GAPDH* near-uniformly performs poorly under the exact same comparisons. Furthermore, all four of our high-scoring candidates show remarkable sample-to-sample RQ agreement (Spearman’s rho values ~0.8), while all four show poor correlation with *GAPDH* (~0.3–0.4) (S8 Table). Given the diverse biochemical roles played by these four genes, a coordinated muscle and disease-associated expression program for all four seems unlikely. Conversely *SDHA*, another key metabolic gene used in our panel, exhibits muscle and disease-specific expression in a manner similar to *GAPDH*, while critically not demonstrating overt sample-to-sample correlation with this gene (Spearman’s rho 0.39). Finally, normalization of data with our four genes resulted in a marked decrease in data spread (lower CoV) in almost all cases, while normalization with *GAPDH* achieved the opposite effect. If one makes the assumption that biological replicates for expression of a given gene should be relatively consistent, one would be forced to conclude our normalization factor serves better in this respect than does *GAPDH*.

## Conclusions

The primary goal of this work was to establish whether a set reference genes exists for mouse muscle that remain suitable for both healthy and dystrophic samples regardless of animal age or muscle type studied: a standard panel of qPCR reference genes that remain valid under essentially all comparative scenarios would, if adopted widely, render multiple studies conducted in multiple research groups highly comparable. As shown here, despite showing small disease-associated changes, the combination of *CSNK2A2*, *AP3D1*, *RPL13a* and *ACTB* appears to fulfil this challenging remit. Moreover, we have previously shown that such a set exists for normalizing expression in healthy and dystrophic canine muscle [20], raising the enticing possibility that a similar set might be identified for use in human patient samples. We would not necessarily expect the same genes to perform well in humans: our data suggest that extrapolation between species is not straightforward. RPL13a performs well in both mouse and dogs (and thus may similarly excel in humans), but our other candidate genes show clear species-specificity. Humans are genetically closer to mice than dogs, but more similar to dogs with respect to muscle metabolism and disease presentation/severity. A human equivalent of the study presented here would be markedly more ambitious, but our data suggests such an effort might yield success.

A secondary goal of this work was to assess the validity of the three most commonly used reference genes in the literature (*GAPDH*, *18S* and *ACTB*), as these three genes have previously been shown to be remarkably poor choices in myogenic cell cultures and dystrophic canine muscle. Unexpectedly, while *GAPDH* and *18S* remain poor candidates in this study, *ACTB* is revealed to be a strong choice in mouse, a finding we hope is of considerable reassurance to many investigators in the *mdx* field. The small dystrophy-associated increase we identify here argues against using this gene alone however, and we would further stress that reliance on any single gene is a risky, error-prone normalization strategy regardless of suitability: in alignment with the MIQE guidelines [16] investigators should always strive to employ two if not three reference genes, especially if attempting to measure subtle changes in expression. We accept that use of all four reference genes suggested here might be considered excessive (though such an approach would be highly rigorous), and our geNorm analysis suggests there is little advantage to be gained in increasing the number of reference genes from three to four (S2 Table). All four genes show very closely-matched expression profiles (S7 and S8 Tables), and while all are high-scoring, no consistent ranking of the four emerges. All four offer advantages with respect to wider comparisons: *CSNK2A2* and *AP3D1* also perform well in myogenic cell cultures, *RPL13a* performs well in dogs, and *ACTB* already enjoys widespread use in *mdx* mice. Given the observations regarding disease-specificity however, we do not recommend pairing *CSNK2A2* and *AP3D1* alone, nor *ACTB* and *RPL13a*, and we thus conclude by suggesting (given the wealth of established literature using *ACTB* as a reference) the most prudent and economical approach for investigators seeking empirically-identified and validated reference genes would be to select *ACTB* first and foremost, and combine it with either *AP3D1* or *CSNK2A2*.

## Supporting information

S1 Appendix Reference gene selection algorithms: Detailed summary. (DOCX)

S2 Appendix Sample summary, Primer and qPCR validation. (DOCX)

S1 Fig Raw Cq data. **(A)** Individual Cq values (**•**) for all 126 samples for each candidate reference gene (as indicated). Genes for which some samples provided no amplicons are indicated, along with number of missing datapoints. **(B)** Relative expression levels for each candidate reference gene in human tissues as reported by the Illumina bodymap project (expression data converted to log(1/expression level) to allow scale-matching with raw Cq data). No bodymap data for 18S exists (mRNA only).
(TIF)

S2 Fig geNorm outputs for all dataset combinations. geNorm ranking by average expression stability M (left to right: least stable to most stable) for the entire dataset, or specific subsets (as indicated). Dashed line: M = 0.5 (threshold of stability for strong candidates).
(TIF)

S3 Fig BestKeeper analysis for all dataset combinations. Coefficient of correlation values for the reference gene candidates are shown for the entire dataset, or specific subsets (as indicated), ranked (left to right) from least stable to most stable.
(TIF)

S4 Fig Normfinder analysis (ungrouped) for all dataset combinations. Stability values (left to right: least stable to most stable) for the reference gene candidates are shown for the entire dataset or specific subsets (as indicated).
(TIF)

S5 Fig Muscle and disease-specific expression patterns of high-scoring candidates. **(A)** Raw RQ values (●) for ACTB, RPL13a, CSNK2A2 and AP3D1 for the entire dataset, separated by healthy/dystrophic. **(B)** Normalized expression data (means + SEM) for ACTB, RPL13a, CSNK2A2 and AP3D1 separated by muscle type and healthy (green) vs dystrophic (red). *:P<0.05, **:P<0.01, ****:P<0.0001, Mann-Whitney U test (healthy/dystrophic). Boxes: Coefficient of Variation.
(TIF)

S6 Fig FBXW2 vs BestKeeper. Raw Cq values for FBXW2 plotted against a BestKeeper derived from the entire dataset (upper panel), or against a BestKeeper derived from the dataset after removal of FBXW2 (lower panel). Boxes: Pearson correlation (r) and significance of correlation.
(TIF)

S1 Table Dataset averages. Arithmetic mean and standard deviations of the Cq values for each candidate gene: of the genes used, FBXW2 shows the greatest sample-to-sample variation, while AP3D1 and CSNK2A2 show the least. Shaded boxes: genes omitted from analysis.
(DOCX)

S2 Table geNorm pairwise variation. Output of the geNorm algorithm for the entire dataset (or subcategory as indicated) showing reduction in pairwise variation with additional reference genes (e.g. V2/3: increasing from 2 -the best pair- to three). Values of 0.2 or lower are considered acceptable, thus three or four reference genes reduce variability but two (the best pair) suffice in all instances.
(DOCX)

S3 Table Age-specific Normfinder rankings (grouped). Normfinder results for age-specific subsets grouped by different criteria (as indicated: top row; datasets, second row; criterion), ranked from highest scoring (lowest stability value) to lowest scoring. Grouped analysis also suggests the best pair of genes for normalization (third row), (not necessarily the highest scoring individually). Bold: stability <0.25; italics: stability > 0.4
(DOCX)

S4 Table Muscle-specific Normfinder rankings (grouped). Normfinder results for muscle-specific subsets grouped by different criteria (as indicated: top row; datasets, second row; criterion), ranked from highest scoring (lowest stability value) to lowest scoring. Grouped analysis also suggests the best pair of genes for normalization (third row), (not necessarily the highest scoring individually). Bold: stability <0.25; italics: stability > 0.4
(DOCX)

S5 Table Age and disease-specific Normfinder rankings (grouped). Normfinder results for age-specific subsets separated by healthy/dystrophic and then grouped by different criteria (as indicated: top row; datasets, second row; criterion), ranked from highest scoring (lowest stability value) to lowest scoring. Grouped analysis also suggests the best pair of genes for normalization (third row), (not necessarily the highest scoring individually). Bold: stability <0.25; italics: stability > 0.4
(DOCX)

S6 Table Normfinder ‘best pair’ combinations for all dataset/grouping combinations. (DOCX)

S7 Table Correlation matrix of Cq values. Pearson correlations (r) for raw Cq values (all genes). Bold: correlations between high scoring candidates (ACTB, RPL13a, CSNK2A2, AP3D1). Italics: correlations with P values greater than 0.0001 (all other correlations P<0.0001); CDC40 vs GAPDH = 0.0026; 18S vs SDHA = 0.0001
(DOCX)

S8 Table Correlation matrix of RQ values. Spearman’s Rho values for RQ correlations (all genes). Bold: correlations between high scoring candidates (ACTB, RPL13a, CSNK2A2, AP3D1). Italics: correlations with P values greater than 0.0001 (all other correlations P<0.0001); CDC40 vs GAPDH = 0.0046; 18S vs SDHA = 0.0002; GAPDH vs B2M = 0.0003.
(DOCX)
