# Supplementary material for: Identification of qPCR reference genes suitable for normalizing gene expression in the mdx mouse model of Duchenne muscular dystrophy
Source: PLoS One. 2019 Jan 30;14(1):e0211384. doi: 10.1371/journal.pone.0211384 (PMC6353192; doi:10.1371/journal.pone.0211384)
Supplement: S1 Appendix — (DOCX) [file pone.0211384.s001.docx]

Reference gene selection algorithms: detailed summary

**geNorm**:

The geNorm algorithm is an iterative methodology, taking the linearised RQ values for each gene (for all cDNA samples) and measuring per-sample pairwise variation of these values with those of all other genes within the dataset. The standard deviations of each full gene-to-gene comparison are averaged for each gene to calculate a gene-specific stability factor (M), essentially a reflection of the extent to which that gene mirrors the individual behaviours of the other genes. The algorithm then discards the lowest scoring candidate (highest M), and repeats the process for the remaining dataset, iterating through this process until only a single pair of highly-correlated genes remain. Under the assumption that the candidate genes used cover a broad range of cellular roles (thus are not co-ordinately regulated), the rationale is that all observed expression variation in the geometric mean of this selected pair consequently reflects the quantity of cDNA present. As a pairwise comparison rather than an assessment of implicit stability, geNorm is thus relatively tolerant of noisy datasets: the changes in expression matter less than the correlation of such changes between genes. The method is concomitantly somewhat sensitive to single extreme sample outliers, as these tend to significantly skew correlation matrices. Additionally, due to the pairwise approach employed, this method will always nominate a pair of genes, and cannot further rank the individual genes forming the best pair: both genes share the same score. Single candidates that are highly stable will be rejected in favour of a pair of less stable candidates with high expression correlation.

The geNorm algorithm has now been folded into the qBase+ program (www.qbaseplus.com), an integrated commercial package that also includes a significant quantity of additional analysis refinements and utilities. For investigators wishing to use the (non-commercial) original excel macro, a copy is hosted at <http://ulozto.net/xsFueHSA/genorm-v3-zip>.

**BestKeeper**:

BestKeeper also employs a pairwise approach, this algorithm instead substituting the iterative strategy of geNorm for a simple comparison. Under the assumption that all genes in the dataset are reasonably strong potential candidates, one can posit that the average of *all* candidate genes should represent a highly-accurate reflection of cDNA content. This method thus generates the geometric mean of the raw Cq values of every gene in the candidate panel to create a single per-sample normalisation factor (the BestKeeper), and then assesses the genes individually for pairwise correlation (Pearson correlation) with this factor, ranking them accordingly. Routine use of ten or more reference genes would be rigorous but highly impractical: this method therefore identifies which genes best mimic the behaviour of the entire dataset as a whole, allowing the investigator to select only those most representative genes. The BestKeeper method also outputs a substantial number of additional metrics (such as individual gene variation) however we have found correlation with the BestKeeper (and matching Pearson correlations for individual genes with each other) to be the most useful assessment. As noted, the method makes the assumption that all genes examined are already comparatively stable, and is thus vulnerable to poor candidate selection (knowing or unknowing). Several highly-disease correlated genes (for example) might skew the BestKeeper value away from identifying more stable candidates. The original write-protected BestKeeper spreadsheet can be obtained from <http://www.gene-quantification.de/bestkeeper.html>, however this file has a hard cap of 10 gene candidates and 100 samples. One could argue that such restrictions are already very generous, but for the entire dataset used in our study (13 gene candidates, 126 samples) we were forced to generate our own equivalent package. Our custom spreadsheet (available on request) does not offer the same refinement, user-friendliness or breadth of metrics as the original, but will calculate the BestKeeper and derive Pearson correlation values for each gene with the BestKeeper (and with all other genes), and will handle up to 20 genes and 400 samples.

**Normfinder**:

Unlike the other two methods, the Normfinder method is not pairwise. Each gene is assessed essentially individually, being consequently ranked by stability (exhibiting the least sample to sample variation across the chosen dataset). A powerful aspect of this algorithm is the capacity for grouped analysis, whereby the stability is assessed both within and between groups specified by the investigator (intra- and inter-group stability). A gene may exhibit considerable variation between samples within each given group, but show no overt variation when groups are compared with each other: such a gene might consequently score highly despite being ostensibly variable. Conversely, a gene might show near-uniform expression within a group, but also show strong group-specific variation (i.e. be uniformly expressed in healthy tissue, and uniformly expressed at a two-fold higher level in diseased tissue). Such a gene would score less highly, and by careful selection of grouping criteria and cross-comparison of results, such a gene could also be identified as disease-associated. A further advantage of this method is the ability to generate a ‘best pair’ (two genes which may not be high scoring individually): by empirically assessing group-specific stability, the Normfinder algorithm can identify a pair of genes that may exhibit substantial intragroup variation, but of opposite signs. In many scenarios no single gene could be expected to be wholly stable, however two genes that vary in equal but opposite fashion can instead be used to generate a highly stable normalisation factor.

Taking full advantage of this method requires large datasets: the minimum number of samples that can constitute a group is necessarily two, and such a small group would be of limited utility. Assessment of intragroup stability concomitantly improves with larger group sizes (and larger datasets). As (in essence) an empirical assessment of noise, the method is also more sensitive to noisy datasets, but less vulnerable to skew from rare outliers.

The Normfinder plugin can be obtained from <http://moma.dk/normfinder-software>
