# Supplementary material for: Identification of qPCR reference genes suitable for normalizing gene expression in the mdx mouse model of Duchenne muscular dystrophy
Source: PLoS One. 2019 Jan 30;14(1):e0211384. doi: 10.1371/journal.pone.0211384 (PMC6353192; doi:10.1371/journal.pone.0211384)
Supplement: S2 Appendix — (DOCX) [file pone.0211384.s002.docx]

Sample summary:

- 3 Age groups (6 weeks, 10 weeks, 24 weeks)
- 6 animals per age group (3 WT, 3 MDX)
- Seven muscles per animal

- 126 samples in total: 18 per muscle, 63 per genotype, 42 per age

**Primer and qPCR validation**:

No DNAse treatment was performed prior to cDNA synthesis: we typically omit this treatment as the DNAse must necessarily be inactivated (typically via heat-treatment) and removed before cDNA synthesis can be performed. Whether via column purification, or solvent extraction and precipitation methods, this additional clean-up step will almost invariably lose or degrade a small fraction of the RNA, consequently introducing an additional source of potential error. In lieu of this process, amplification of genomic DNA (gDNA) can instead be avoided by design of primers to span large introns, and the pan-specific primers to *HPRT1* (taken from [1]) were designed to span one such intron while also providing a high level of mismatch against *HPRT1* pseudogenes. No sequences are provided for primers from the PrimerDesign geNorm and geNormPLUS sets, thus we cannot confirm whether these necessarily span introns: instead the anchor nucleotides and context sequence lengths (as suggested by [2]) are provided below.

| **Official gene symbol** | **Accession number** | **Anchor Nucleotide** | **Context length sequence (bp)** |
| --- | --- | --- | --- |
| ***Cdc40*** | NM_027879 | 488 | 167 |
| ***Ap3d1*** | NM_007460 | 2694 | 183 |
| ***Zfp91*** | NM_053009 | 1415 | 148 |
| ***ACTB*** | NM_007393.3 | 597 | 94 |
| ***GAPDH*** | NM_008084.2 | 793 | 180 |
| ***Csnk2a2*** | NM_009974 | 1086 | 177 |
| ***B2M*** | NM_009735.3 | 202 | 159 |
| ***Fbxo38*** | NM_134136 | 1797 | 172 |
| ***Mon2*** | NM_001163024 | 4707 | 166 |
| ***Htatsf1*** | NM_028242 | 1259 | 136 |
| ***Fbxw2*** | NM_013890 | 655 | 206 |
| ***Pak1ip1*** | NM_026550 | 737 | 114 |
| ***SDHA*** | NM_023281.1 | 2018 | 181 |
| ***RPL13A*** | NM_009438.5 | 691 | 180 |
| ***18S*** | NR_003278.3 | 134 | 99 |

*Context length sequences and anchor nucleotides for the amplicons used in this study*.

To establish whether trace gDNA contamination (if present) could plausibly influence measured Cq values, all primers were used in qPCR with a subset of cDNA samples prepared without reverse transcriptase (-RT). As shown below, 9 of the 16 primer pairs gave no product when reverse transcriptase was omitted from cDNA synthesis, while the remaining 7 gave Cq values 6-10 cycles greater than that observed when reverse transcriptase was included, showing that genomic DNA was indeed present, but at levels safely below the threshold of influence (contamination at a level detectable after an additional 6 cycles corresponds to a ~64-fold reduction in signal: the effect of such contamination on measured Cq values amounts to approximately 0.022 cycles, considerably lower than the innate variation between replicate wells). Note that both 18S and GAPDH gave relatively low Cq values even for trace gDNA contamination, reflecting the high copy number of the rRNA locus in the case of 18S and the large number of genomic pseudogenes in the case of GAPDH.

| Gene | Mean Cq +RT | StDev | Mean Cq -RT | StDev |
| --- | --- | --- | --- | --- |
| *cdc40* | 25.11 | 1.065 | N/D | - |
| *Ap3d1* | 23.4 | 0.883 | N/D | - |
| *Zfp91* | 23.22 | 2.675 | N/D | - |
| *ActB* | 19.91 | 1.076 | 29.02 | - |
| *GAPDH* | 15.91 | 1.245 | 24.49 | 0.571 |
| *Csnk2a2* | 25.54 | 0.825 | N/D | - |
| *B2M* | 18.01 | 1.124 | 30.9 | 0.73 |
| *Fbxo38* | 24.28 | 1.837 | N/D | - |
| *Mon2* | 22.99 | 1.942 | N/D | - |
| *Htatsf1* | 23 | 1.006 | 33.34 | 0.224 |
| *Fbxw2* | 26.73 | 1.685 | N/D | - |
| *Pak1ip1* | 22.58 | 0.986 | N/D | - |
| *SDHA* | 19.01 | 1.202 | 29.48 | 0.298 |
| *RPL13a* | 24.69 | 0.979 | 30.91 | 0.545 |
| *18S* | 9.62 | 1.253 | 24.67 | 0.028 |
| *HPRT1* | 24.15 | 1.248 | N/D | - |

*Average Cq values (and standard deviations) for cDNA prepared with and without reverse transcriptase.*

Mean Cq values for +RT were obtained from the entire dataset (N=121-126) while –RT controls used a small selection of samples (N=3) but revealed a relatively consistent (low) level of gDNA.

**Melt curves:**

All melt curves shown a single prominent amplicon with melt peaks of between 78 and 85°C: typical replicate traces are shown below.


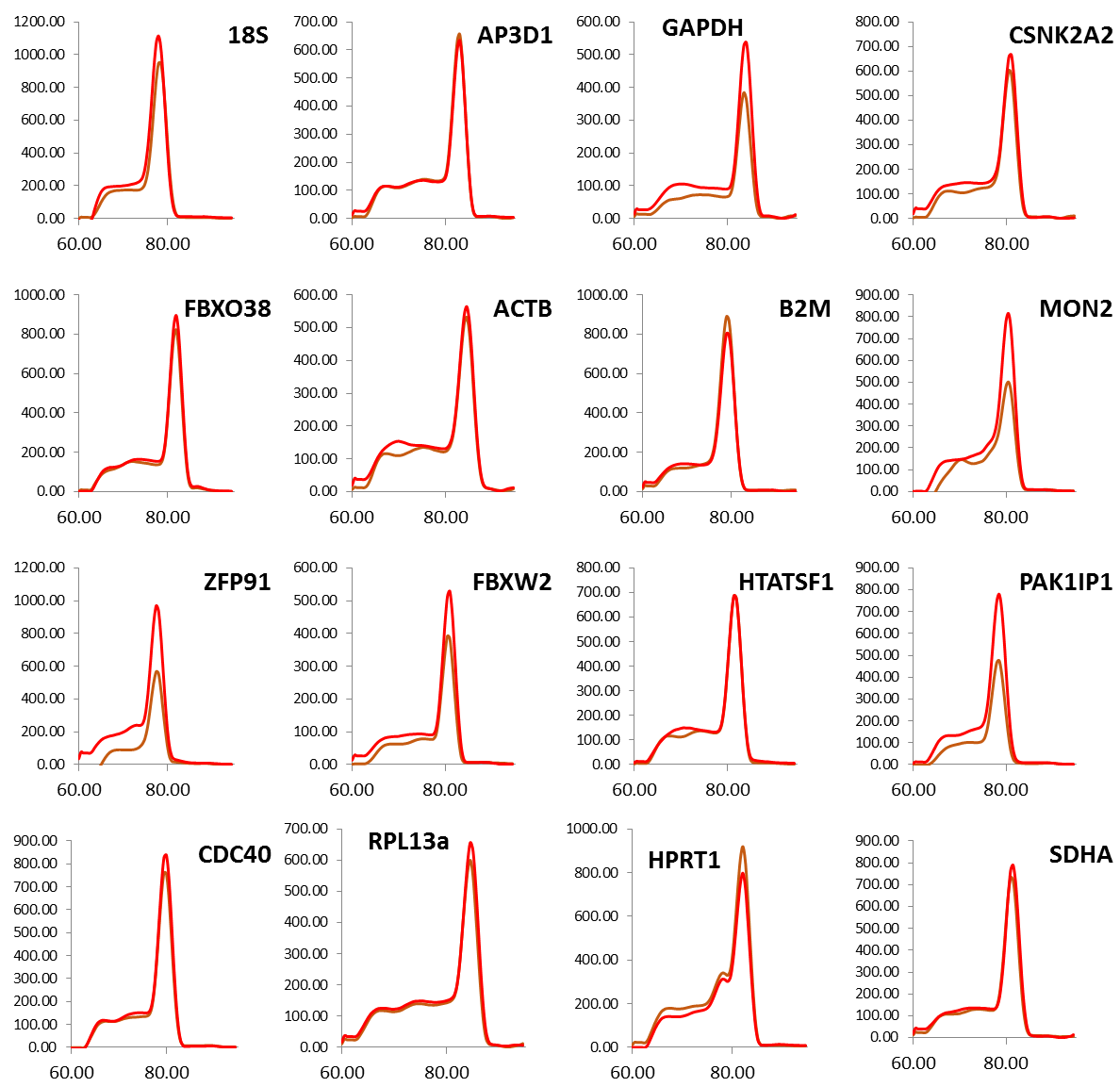


Typical melt curves for the 16 primer pairs used in this study: two representative traces are shown for each gene.

[1] Valadan R, Hedayatizadeh-Omran A, Alhosseini-Abyazani MN*, et al.* Data supporting the design and evaluation of a universal primer pair for pseudogene-free amplification of HPRT1 in real-time PCR. Data in Brief. 2015;4:384-9. doi: 10.1016/j.dib.2015.06.009

[2] Bustin SA, Benes V, Garson JA*, et al.* Primer Sequence Disclosure: A Clarification of the MIQE Guidelines. Clinical chemistry. 2011;57(6):919-21. doi: 10.1373/clinchem.2011.162958
