# Supplementary figures and images for: Identification of qPCR reference genes suitable for normalizing gene expression in the mdx mouse model of Duchenne muscular dystrophy
Source: PLoS One. 2019 Jan 30;14(1):e0211384. doi: 10.1371/journal.pone.0211384 (PMC6353192; doi:10.1371/journal.pone.0211384)

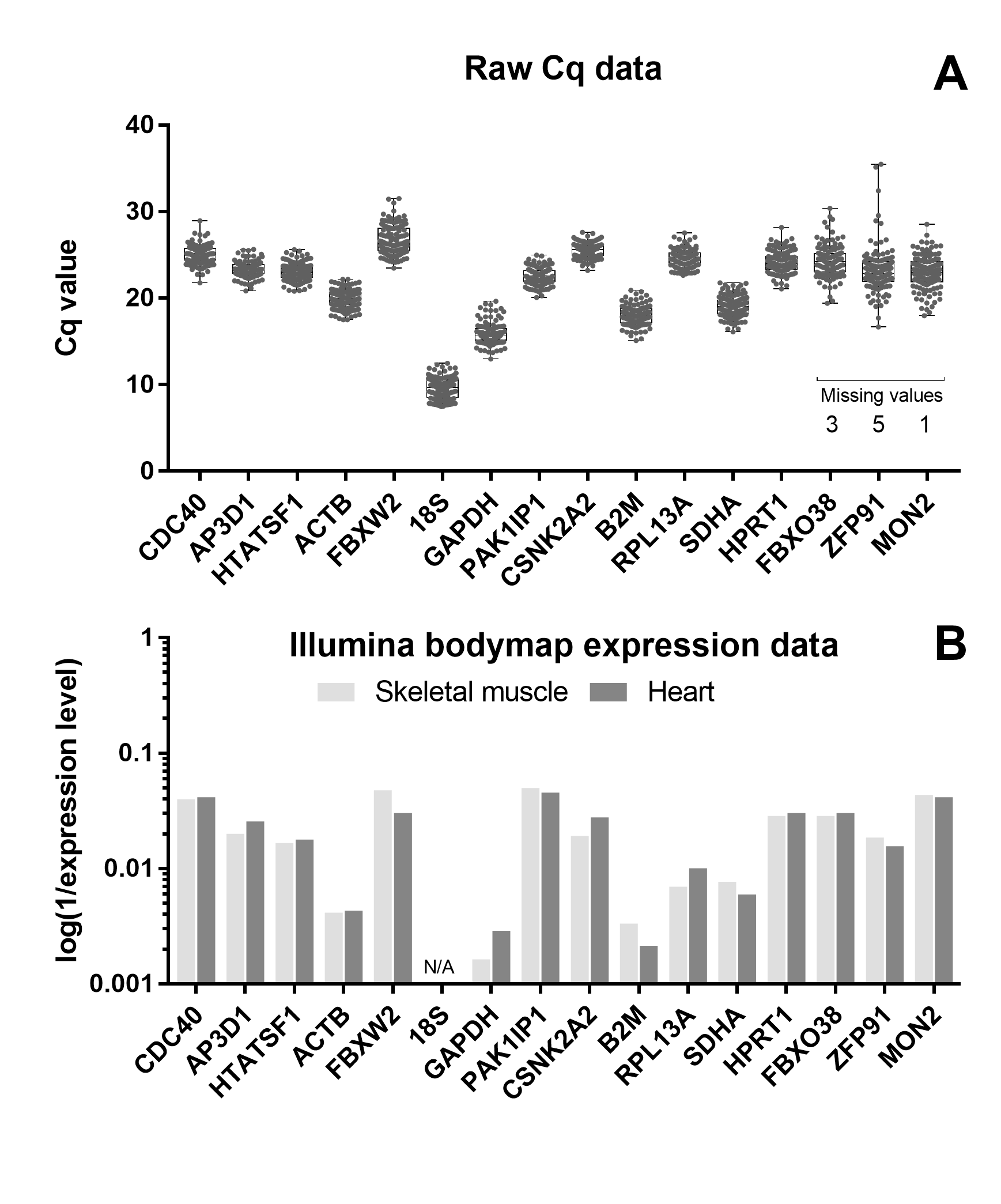

Supplement: S1 Fig — (A) Individual Cq values (•) for all 126 samples for each candidate reference gene (as indicated). Genes for which some samples provided no amplicons are indicated, along with number of missing datapoints. (B) Relative expression levels for each candidate reference gene in human tissues as reported by the Illumina bodymap project (expression data converted to log(1/expression level) to allow scale-matching with raw Cq data). No bodymap data for 18S exists (mRNA only). (TIF) [file pone.0211384.s003.tif]

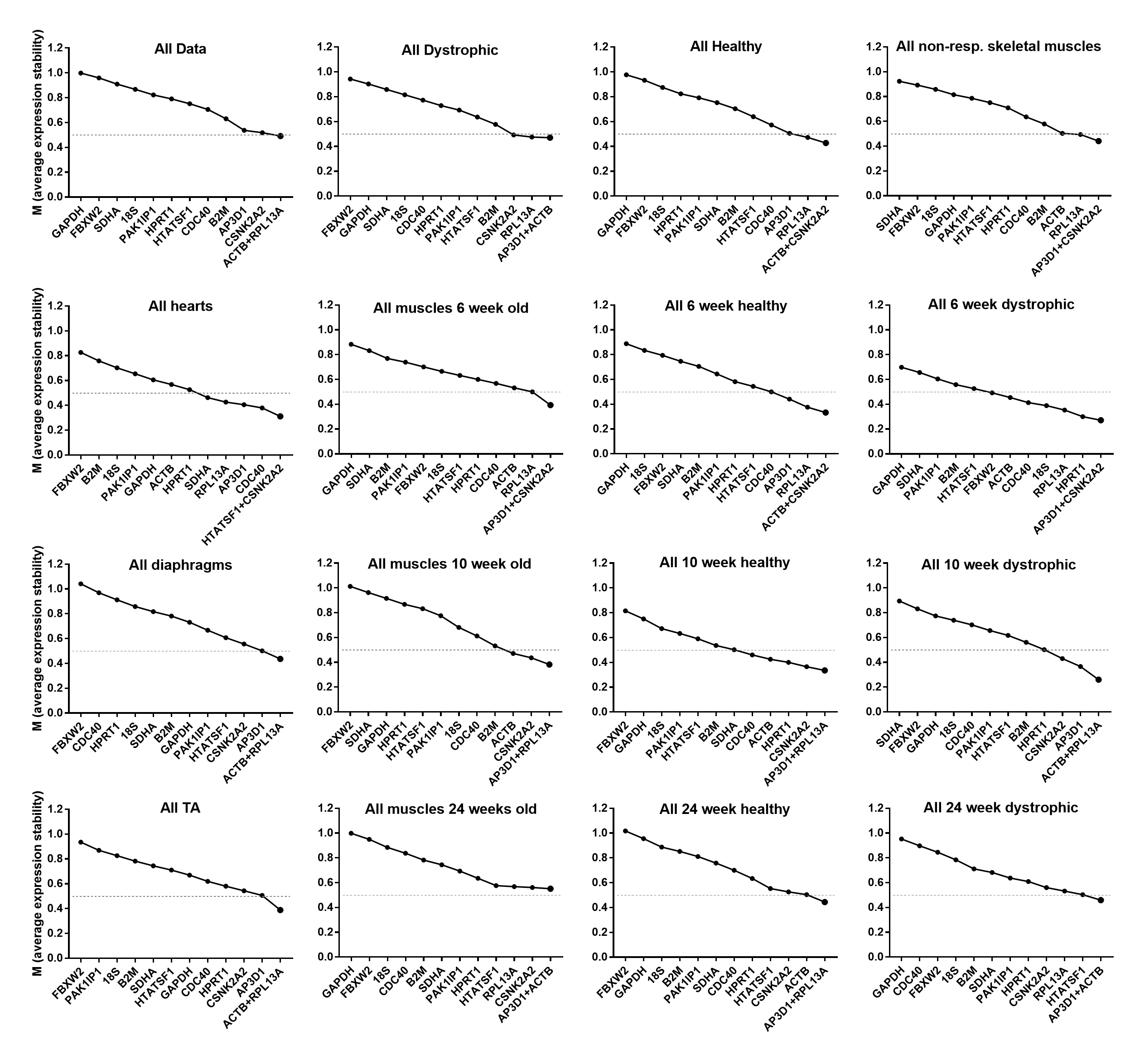

Supplement: S2 Fig — geNorm ranking by average expression stability M (left to right: least stable to most stable) for the entire dataset, or specific subsets (as indicated). Dashed line: M = 0.5 (threshold of stability for strong candidates). (TIF) [file pone.0211384.s004.tif]

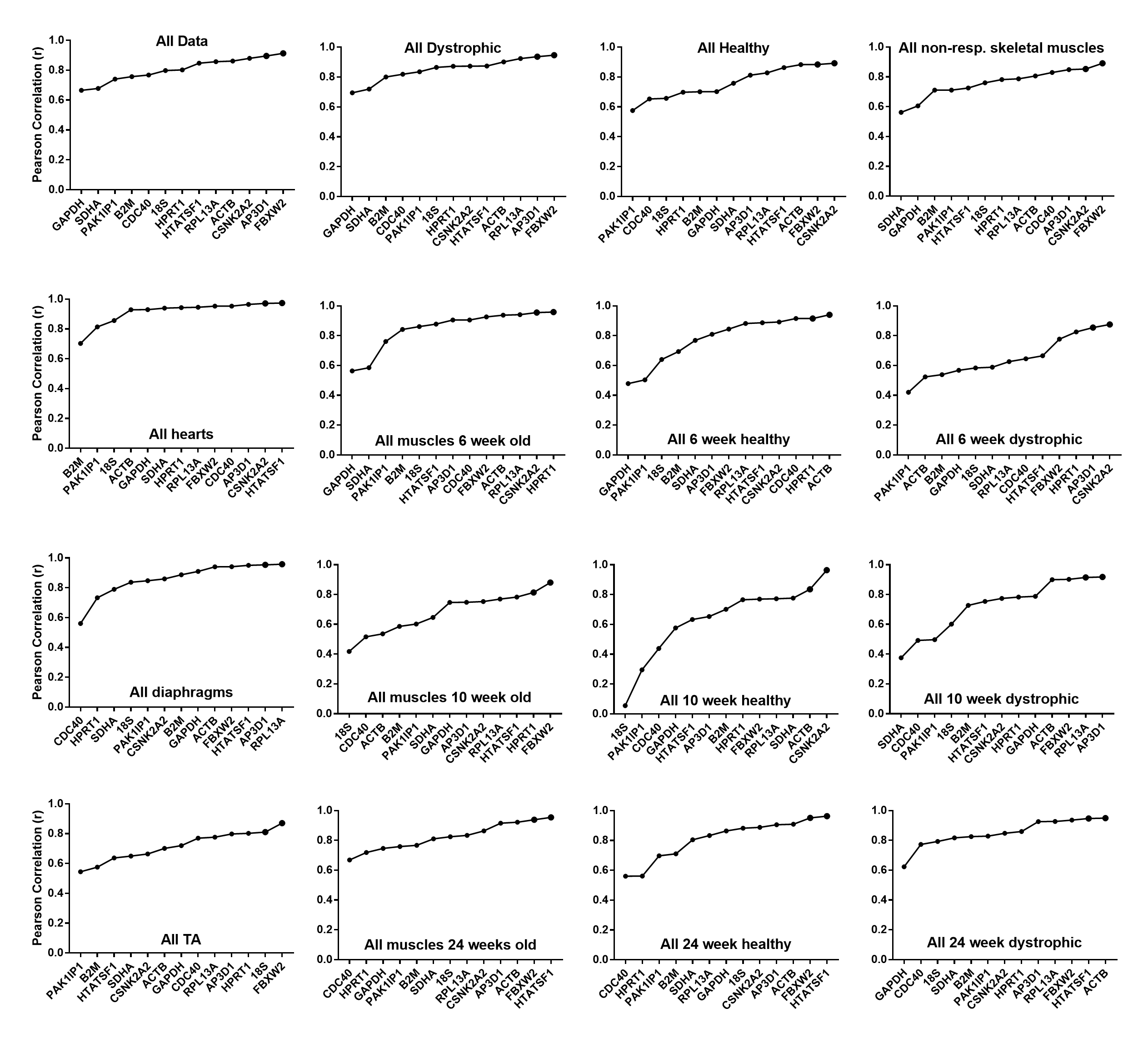

Supplement: S3 Fig — Coefficient of correlation values for the reference gene candidates are shown for the entire dataset, or specific subsets (as indicated), ranked (left to right) from least stable to most stable. (TIF) [file pone.0211384.s005.tif]

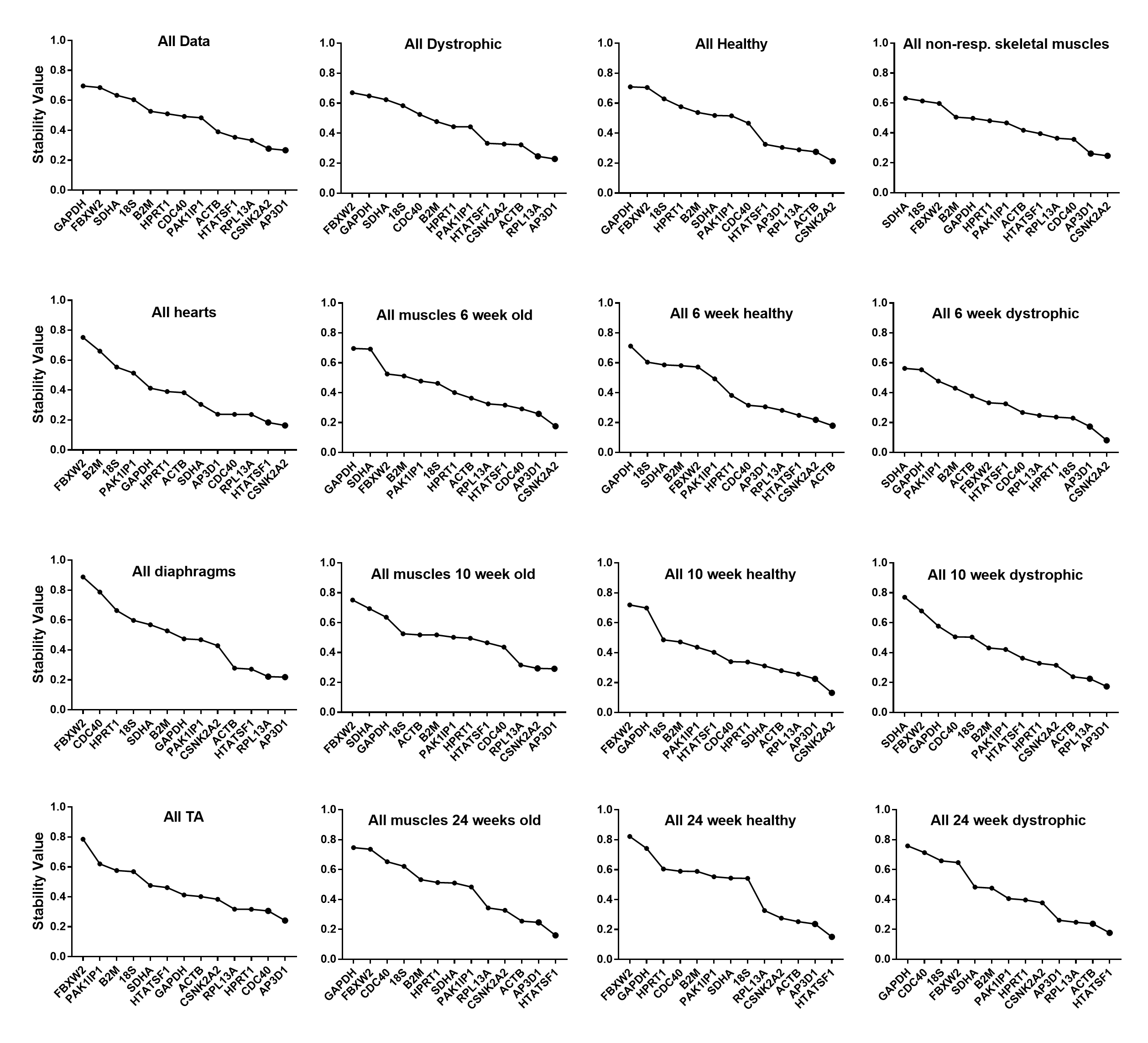

Supplement: S4 Fig — Stability values (left to right: least stable to most stable) for the reference gene candidates are shown for the entire dataset or specific subsets (as indicated). (TIF) [file pone.0211384.s006.tif]

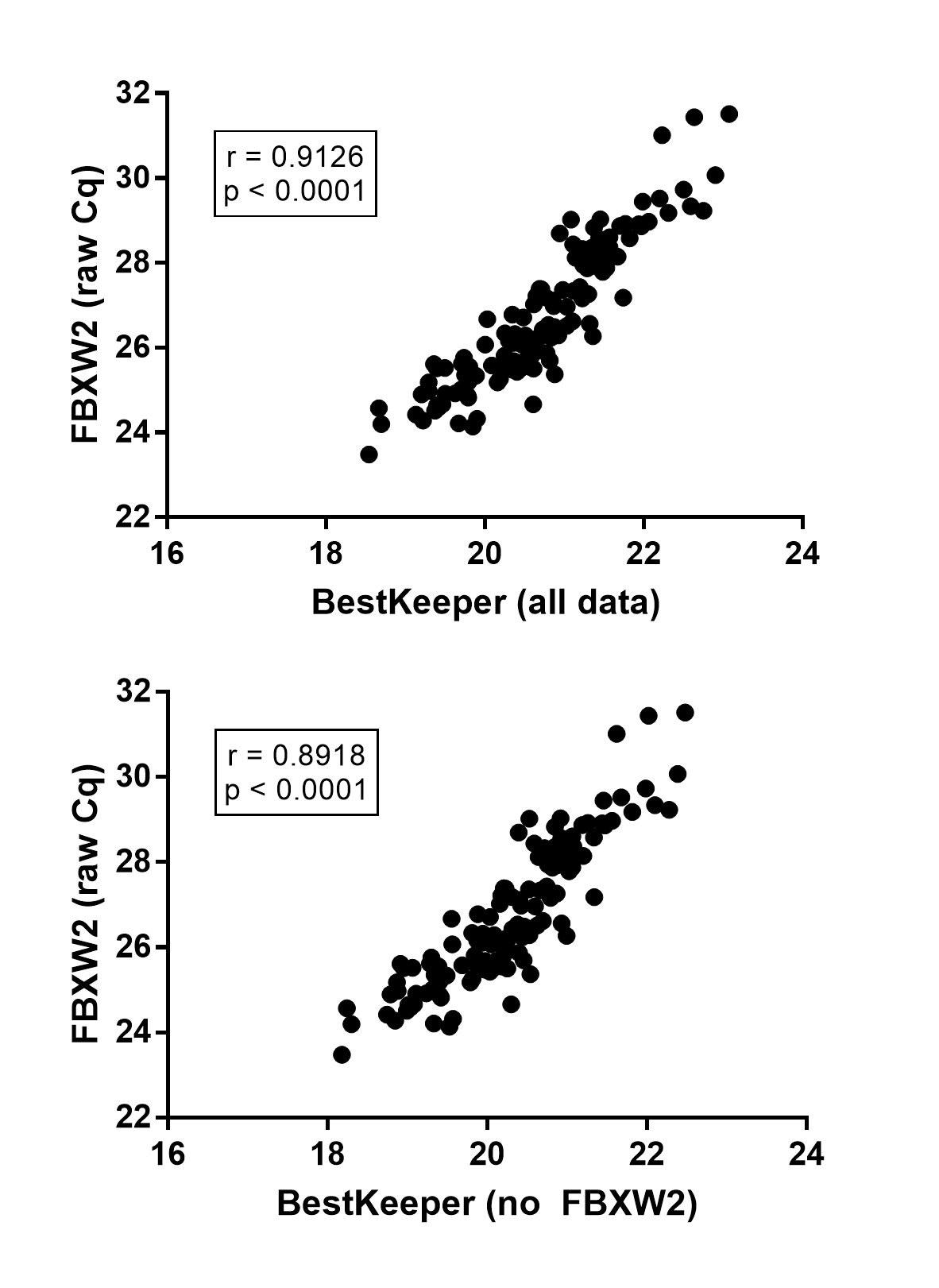

Supplement: S6 Fig — Raw Cq values for FBXW2 plotted against a BestKeeper derived from the entire dataset (upper panel), or against a BestKeeper derived from the dataset after removal of FBXW2 (lower panel). Boxes: Pearson correlation (r) and significance of correlation. (TIF) [file pone.0211384.s008.tif]
