# Supplementary material for: Identification of qPCR reference genes suitable for normalizing gene expression in the mdx mouse model of Duchenne muscular dystrophy
Source: PLoS One. 2019 Jan 30;14(1):e0211384. doi: 10.1371/journal.pone.0211384 (PMC6353192; doi:10.1371/journal.pone.0211384)
Supplement: S1 Table — Arithmetic mean and standard deviations of the Cq values for each candidate gene: of the genes used, FBXW2 shows the greatest sample-to-sample variation, while AP3D1 and CSNK2A2 show the least. Shaded boxes: genes omitted from analysis. (DOCX) [file pone.0211384.s009.docx]

|  | **CDC40** | **AP3D1** | **HTATSF1** | **ACTB** | **FBXW2** | **18S** | **GAPDH** | **PAK1IP1** | **CSNK2A2** | **B2M** | **RPL13A** | **SDHA** | **HPRT1** | **FBXO38** | **ZFP91** | **MON2** |
| --- | --- | --- | --- | --- | --- | --- | --- | --- | --- | --- | --- | --- | --- | --- | --- | --- |
| Mean Cq | 25.1 | 23.4 | 23.0 | 19.9 | 26.7 | 9.6 | 15.9 | 22.6 | 25.5 | 18.0 | 24.7 | 19.0 | 24.1 | 24.3 | 23.2 | 23.0 |
| S.D. | 1.1 | 0.9 | 1.0 | 1.1 | 1.7 | 1.3 | 1.2 | 1.0 | 0.8 | 1.1 | 1.0 | 1.2 | 1.2 | 1.8 | 2.7 | 1.9 |
