# Supplementary material for: Identification of qPCR reference genes suitable for normalizing gene expression in the mdx mouse model of Duchenne muscular dystrophy
Source: PLoS One. 2019 Jan 30;14(1):e0211384. doi: 10.1371/journal.pone.0211384 (PMC6353192; doi:10.1371/journal.pone.0211384)
Supplement: S2 Table — Output of the geNorm algorithm for the entire dataset (or subcategory as indicated) showing reduction in pairwise variation with additional reference genes (e.g. V2/3: increasing from 2 -the best pair- to three). Values of 0.2 or lower are considered acceptable, thus three or four reference genes reduce variability but two (the best pair) suffice in all instances. (DOCX) [file pone.0211384.s010.docx]

|  | All data | All healthy | All DMD | All 6wk | All 10wk | All 24wk | All skeletal muscle | All DIA | All Hearts | All TA | 6wk healthy | 6wk DMD | 10wk healthy | 10wk DMD | 24wk healthy | 24wk DMD |
| --- | --- | --- | --- | --- | --- | --- | --- | --- | --- | --- | --- | --- | --- | --- | --- | --- |
| V2/3 | 0.16 | 0.15 | 0.14 | 0.17 | 0.14 | 0.17 | 0.16 | 0.16 | 0.13 | 0.18 | 0.12 | 0.09 | 0.11 | 0.13 | 0.16 | 0.16 |
| V3/4 | 0.12 | 0.12 | 0.11 | 0.12 | 0.11 | 0.12 | 0.11 | 0.14 | 0.09 | 0.13 | 0.11 | 0.09 | 0.10 | 0.11 | 0.12 | 0.12 |
| V4/5 | 0.14 | 0.12 | 0.13 | 0.11 | 0.11 | 0.10 | 0.12 | 0.12 | 0.08 | 0.11 | 0.10 | 0.08 | 0.08 | 0.11 | 0.10 | 0.10 |
| V5/6 | 0.13 | 0.11 | 0.11 | 0.09 | 0.12 | 0.11 | 0.11 | 0.12 | 0.08 | 0.10 | 0.09 | 0.07 | 0.08 | 0.10 | 0.12 | 0.10 |
| V6/7 | 0.11 | 0.11 | 0.10 | 0.09 | 0.11 | 0.10 | 0.11 | 0.11 | 0.09 | 0.10 | 0.08 | 0.07 | 0.08 | 0.10 | 0.11 | 0.08 |
| V7/8 | 0.10 | 0.10 | 0.09 | 0.08 | 0.12 | 0.10 | 0.09 | 0.10 | 0.08 | 0.09 | 0.09 | 0.07 | 0.07 | 0.08 | 0.10 | 0.09 |
| V8/9 | 0.09 | 0.09 | 0.09 | 0.08 | 0.10 | 0.09 | 0.08 | 0.09 | 0.07 | 0.08 | 0.09 | 0.06 | 0.08 | 0.08 | 0.10 | 0.08 |
| V9/10 | 0.09 | 0.08 | 0.08 | 0.08 | 0.08 | 0.09 | 0.08 | 0.09 | 0.07 | 0.08 | 0.08 | 0.06 | 0.07 | 0.08 | 0.09 | 0.10 |
| V10/11 | 0.09 | 0.09 | 0.08 | 0.07 | 0.09 | 0.09 | 0.08 | 0.09 | 0.07 | 0.08 | 0.08 | 0.06 | 0.07 | 0.07 | 0.08 | 0.09 |
| V11/12 | 0.09 | 0.09 | 0.08 | 0.09 | 0.09 | 0.09 | 0.07 | 0.09 | 0.08 | 0.08 | 0.08 | 0.07 | 0.09 | 0.08 | 0.10 | 0.08 |
| V12/13 | 0.08 | 0.08 | 0.08 | 0.08 | 0.08 | 0.08 | 0.07 | 0.10 | 0.08 | 0.09 | 0.08 | 0.06 | 0.08 | 0.09 | 0.09 | 0.09 |
