# Supplementary material for: Identification of qPCR reference genes suitable for normalizing gene expression in the mdx mouse model of Duchenne muscular dystrophy
Source: PLoS One. 2019 Jan 30;14(1):e0211384. doi: 10.1371/journal.pone.0211384 (PMC6353192; doi:10.1371/journal.pone.0211384)
Supplement: S3 Table — Normfinder results for age-specific subsets grouped by different criteria (as indicated: top row; datasets, second row; criterion), ranked from highest scoring (lowest stability value) to lowest scoring. Grouped analysis also suggests the best pair of genes for normalization (third row), (not necessarily the highest scoring individually). Bold: stability <0.25; italics: stability > 0.4 (DOCX) [file pone.0211384.s011.docx]

|  | All 6 week data | | | All 10 week data | | | All 24 week data | | |
| --- | --- | --- | --- | --- | --- | --- | --- | --- | --- |
|  | Animal | Disease | Muscle | Animal | Disease | Muscle | Animal | Disease | Muscle |
| Best pair | **HTATSF1**  **+**  **RPL13a** | **18S**  **+**  **CSNK2A2** | **CDC40**  **+**  **CSNK2A2** | **AP3D1**  **+**  **HTATSF1** | **AP3D1**  **+**  **HTATSF1** | **AP3D1**  **+**  **CSNK2A2** | **AP3D1**  **+**  **HTATSF1** | **AP3D1**  **+**  **HTATSF1** | **AP3D1**  **+**  **HTATSF1** |
| Most | **CSNK2A2** | **CSNK2A2** | **CSNK2A2** | CSNK2A2 | **CDC40** | **CSNK2A2** | **HTATSF1** | **HTATSF1** | **HTATSF1** |
| stable | **AP3D1** | **CDC40** | **CDC40** | AP3D1 | CSNK2A2 | **RPL13A** | **AP3D1** | **AP3D1** | **AP3D1** |
|  | **CDC40** | **AP3D1** | **AP3D1** | CDC40 | AP3D1 | **AP3D1** | **ACTB** | **CSNK2A2** | **ACTB** |
|  | **HTATSF1** | **PAK1IP1** | HTATSF1 | RPL13A | RPL13A | HPRT1 | **RPL13A** | **ACTB** | **CSNK2A2** |
|  | 18S | **HTATSF1** | RPL13A | 18S | GAPDH | PAK1IP1 | **CSNK2A2** | **SDHA** | RPL13A |
|  | RPL13A | **18S** | ACTB | GAPDH | 18S | B2M | **SDHA** | **PAK1IP1** | B2M |
|  | PAK1IP1 | **B2M** | HPRT1 | HTATSF1 | HTATSF1 | ACTB | **PAK1IP1** | **B2M** | PAK1IP1 |
|  | ACTB | RPL13A | 18S | PAK1IP1 | PAK1IP1 | HTATSF1 | **HPRT1** | **HPRT1** | HPRT1 |
|  | B2M | ACTB | PAK1IP1 | *B2M* | B2M | CDC40 | **B2M** | **CDC40** | SDHA |
|  | HPRT1 | HPRT1 | B2M | *HPRT1* | *HPRT1* | 18S | **CDC40** | **RPL13A** | *CDC40* |
|  | FBXW2 | FBXW2 | FBXW2 | *ACTB* | *FBXW2* | FBXW2 | **18S** | **18S** | *18S* |
| Least | GAPDH | GAPDH | *GAPDH* | *SDHA* | *SDHA* | *SDHA* | FBXW2 | **FBXW2** | *FBXW2* |
| stable | *SDHA* | *SDHA* | *SDHA* | *FBXW2* | *ACTB* | *GAPDH* | GAPDH | **GAPDH** | *GAPDH* |
|  |  |  |  |  |  |  |  |  |  |
