# Supplementary material for: Identification of qPCR reference genes suitable for normalizing gene expression in the mdx mouse model of Duchenne muscular dystrophy
Source: PLoS One. 2019 Jan 30;14(1):e0211384. doi: 10.1371/journal.pone.0211384 (PMC6353192; doi:10.1371/journal.pone.0211384)
Supplement: S4 Table — Normfinder results for muscle-specific subsets grouped by different criteria (as indicated: top row; datasets, second row; criterion), ranked from highest scoring (lowest stability value) to lowest scoring. Grouped analysis also suggests the best pair of genes for normalization (third row), (not necessarily the highest scoring individually). Bold: stability <0.25; italics: stability > 0.4 (DOCX) [file pone.0211384.s012.docx]

|  | Non-respiratory skeletal muscle | | | | Diaphragm only | | Heart only | | TA only | |
| --- | --- | --- | --- | --- | --- | --- | --- | --- | --- | --- |
|  | Animal | Disease | Muscle | Age | Disease | Age | Disease | Age | Disease | Age |
| Best pair | **AP3D1**  **+**  **HTATSF1** | **CDC40**  **+**  **AP3D1** | **AP3D1**  **+**  **CSNK2A2** | **AP3D1**  **+**  **CSNK2A2** | **AP3D1**  **+**  **HTATSF1** | **AP3D1**  **+**  **HTATSF1** | **HTATSF1+**  **CSNK2A2** | **AP3D1**  **+**  **CSNK2A2** | **RPL13A**  **+**  **SDHA** | **CDC40**  **+**  **AP3D1** |
| Most | **CSNK2A2** | **CSNK2A2** | **CSNK2A2** | **CSNK2A2** | **AP3D1** | **RPL13A** | **CSNK2A2** | **CSNK2A2** | **AP3D1** | **AP3D1** |
| stable | AP3D1 | **AP3D1** | **CDC40** | **AP3D1** | **HTATSF1** | **AP3D1** | **HTATSF1** | **HTATSF1** | **CDC40** | **CDC40** |
|  | CDC40 | **CDC40** | **AP3D1** | **ACTB** | **RPL13A** | **ACTB** | **AP3D1** | **AP3D1** | **CSNK2A2** | **HPRT1** |
|  | HTATSF1 | **HPRT1** | **ACTB** | **RPL13A** | **PAK1IP1** | HTATSF1 | **CDC40** | **CDC40** | **HPRT1** | **RPL13A** |
|  | RPL13A | **18S** | **B2M** | **PAK1IP1** | **CSNK2A2** | PAK1IP1 | **RPL13A** | **RPL13A** | 18S | **ACTB** |
|  | PAK1IP1 | **PAK1IP1** | **PAK1IP1** | **SDHA** | **ACTB** | CSNK2A2 | **SDHA** | **SDHA** | RPL13A | **CSNK2A2** |
|  | ACTB | **FBXW2** | **HTATSF1** | **B2M** | B2M | *GAPDH* | **HPRT1** | **GAPDH** | GAPDH | **HTATSF1** |
|  | *HPRT1* | HTATSF1 | **GAPDH** | **GAPDH** | GAPDH | *CDC40* | **ACTB** | **HPRT1** | FBXW2 | **GAPDH** |
|  | *GAPDH* | GAPDH | **HPRT1** | **HTATSF1** | CDC40 | *SDHA* | **GAPDH** | **ACTB** | HTATSF1 | **SDHA** |
|  | *B2M* | RPL13A | **RPL13A** | **CDC40** | HPRT1 | *B2M* | **PAK1IP1** | **PAK1IP1** | ACTB | **B2M** |
|  | *FBXW2* | ACTB | **FBXW2** | **HPRT1** | 18S | *HPRT1* | **18S** | **18S** | PAK1IP1 | **PAK1IP1** |
| Least | *18S* | B2M | **18S** | **FBXW2** | FBXW2 | *18S* | **B2M** | B2M | *SDHA* | **18S** |
| stable | *SDHA* | SDHA | **SDHA** | 18S | SDHA | *FBXW2* | FBXW2 | FBXW2 | *B2M* | FBXW2 |
|  |  |  |  |  |  |  |  |  |  |  |
