# Supplementary material for: Identification of qPCR reference genes suitable for normalizing gene expression in the mdx mouse model of Duchenne muscular dystrophy
Source: PLoS One. 2019 Jan 30;14(1):e0211384. doi: 10.1371/journal.pone.0211384 (PMC6353192; doi:10.1371/journal.pone.0211384)
Supplement: S5 Table — Normfinder results for age-specific subsets separated by healthy/dystrophic and then grouped by different criteria (as indicated: top row; datasets, second row; criterion), ranked from highest scoring (lowest stability value) to lowest scoring. Grouped analysis also suggests the best pair of genes for normalization (third row), (not necessarily the highest scoring individually). Bold: stability <0.25; italics: stability > 0.4 (DOCX) [file pone.0211384.s013.docx]

|  | All 6 week data (healthy) | | All 10 week data (healthy) | | All 24 week data (healthy) | | All 6 week data (dystrophic) | | All 10 week data (dystrophic) | | All 24 week data (dystrophic) | |
| --- | --- | --- | --- | --- | --- | --- | --- | --- | --- | --- | --- | --- |
|  | Animal | Muscle | Animal | Muscle | Animal | Muscle | Animal | Muscle | Animal | Muscle | Animal | Muscle |
| Best pair | **ACTB**  **+**  **CSNK2A2** | **HTATSF1**  **+**  **ACTB** | **AP3D1**  **+**  **CSNK2A2** | **AP3D1**  **+**  **CSNK2A2** | **AP3D1**  **+**  **HTATSF1** | **AP3D1**  **+**  **HTATSF1** | **AP3D1**  **+**  **CSNK2A2** | **AP3D1**  **+**  **CSNK2A2** | **AP3D1**  **+**  **B2M** | **AP3D1**  **+**  **CSNK2A2** | **AP3D1**  **+**  **HTATSF1** | **HTATSF1**  **+**  **CSNK2A2** |
| Most | **ACTB** | **ACTB** | **CSNK2A2** | **CSNK2A2** | **HTATSF1** | **HTATSF1** | **CSNK2A2** | **CSNK2A2** | **AP3D1** | **AP3D1** | **HTATSF1** | **HTATSF1** |
| stable | **CSNK2A2** | CSNK2A2 | **AP3D1** | **AP3D1** | **AP3D1** | **AP3D1** | **AP3D1** | **AP3D1** | **RPL13A** | **ACTB** | **AP3D1** | **RPL13A** |
|  | **RPL13A** | RPL13A | **ACTB** | **ACTB** | **ACTB** | CSNK2A2 | **18S** | **HPRT1** | **HPRT1** | RPL13A | **RPL13A** | CSNK2A2 |
|  | **HTATSF1** | CDC40 | **RPL13A** | RPL13A | **CSNK2A2** | ACTB | **CDC40** | **18S** | **ACTB** | HPRT1 | **ACTB** | ACTB |
|  | **CDC40** | HTATSF1 | **CDC40** | CDC40 | **RPL13A** | RPL13A | **RPL13A** | CDC40 | **HTATSF1** | CSNK2A2 | **CSNK2A2** | AP3D1 |
|  | **AP3D1** | AP3D1 | **SDHA** | SDHA | **CDC40** | *CDC40* | **HPRT1** | RPL13A | **CSNK2A2** | PAK1IP1 | **HPRT1** | PAK1IP1 |
|  | **HPRT1** | HPRT1 | **HPRT1** | HPRT1 | **PAK1IP1** | *HPRT1* | **HTATSF1** | FBXW2 | **PAK1IP1** | HTATSF1 | **SDHA** | HPRT1 |
|  | **PAK1IP1** | PAK1IP1 | **HTATSF1** | HTATSF1 | **SDHA** | *B2M* | **FBXW2** | HTATSF1 | **CDC40** | B2M | **B2M** | *SDHA* |
|  | **FBXW2** | *18S* | **B2M** | PAK1IP1 | **B2M** | *PAK1IP1* | **ACTB** | ACTB | **B2M** | CDC40 | **PAK1IP1** | *B2M* |
|  | **B2M** | *FBXW2* | **PAK1IP1** | *B2M* | **18S** | *18S* | **PAK1IP1** | B2M | **18S** | *18S* | **CDC40** | *CDC40* |
|  | **18S** | *SDHA* | **18S** | *18S* | **HPRT1** | *SDHA* | **B2M** | *PAK1IP1* | **GAPDH** | *FBXW2* | **FBXW2** | *18S* |
| Least | **SDHA** | *B2M* | **FBXW2** | *FBXW2* | GAPDH | *FBXW2* | **GAPDH** | *GAPDH* | FBXW2 | *GAPDH* | 18S | *FBXW2* |
| stable | GAPDH | *GAPDH* | GAPDH | *GAPDH* | FBXW2 | *GAPDH* | **SDHA** | *SDHA* | SDHA | *SDHA* | GAPDH | *GAPDH* |
