# Supplementary material for: Identification of qPCR reference genes suitable for normalizing gene expression in the mdx mouse model of Duchenne muscular dystrophy
Source: PLoS One. 2019 Jan 30;14(1):e0211384. doi: 10.1371/journal.pone.0211384 (PMC6353192; doi:10.1371/journal.pone.0211384)
Supplement: S6 Table — (DOCX) [file pone.0211384.s014.docx]

|  | All data | All healthy | All DMD | All 6wk | All 10wk | All 24wk | All skel muscle | All DIA | All heart | All TA | 6wk healthy | 10wk healthy | 24wk healthy | 6wk DMD | 10wk DMD | 24wk DMD |
| --- | --- | --- | --- | --- | --- | --- | --- | --- | --- | --- | --- | --- | --- | --- | --- | --- |
| Animal | AP3D1  +  HTATSF1 | HTATSF1 + CSNK2A2 | PAK1IP1 + RPL13A | HTATSF1 + RPL13A | AP3D1  + HTATSF1 | AP3D1  + HTATSF1 | AP3D1  + HTATSF1 | - | - | - | ACTB  + CSNK2A2 | AP3D1  + CSNK2A2 | AP3D1  + HTATSF1 | AP3D1  + CSNK2A2 | AP3D1  +  B2M | AP3D1  + HTATSF1 |
| Disease | HTATSF1 +  B2M | - | - | 18S  + CSNK2A2 | AP3D1  + HTATSF1 | AP3D1  + HTATSF1 | CDC40  +  AP3D1 | AP3D1  + HTATSF1 | HTATSF1 + CSNK2A2 | RPL13A +  SDHA | - | - | - | - | - | - |
| Muscle | AP3D1  + HTATSF1 | AP3D1  + HTATSF1 | AP3D1  + CSNK2A2 | CDC40  + CSNK2A2 | AP3D1  + CSNK2A2 | AP3D1  + HTATSF1 | AP3D1  + CSNK2A2 | - | - | - | HTATSF1  +  ACTB | AP3D1  + CSNK2A2 | AP3D1  + HTATSF1 | AP3D1  + CSNK2A2 | AP3D1  + CSNK2A2 | HTATSF1 + CSNK2A2 |
| Age | AP3D1  + CSNK2A2 | AP3D1  + HTATSF1 | AP3D1  + PAK1IP1 | - | - | - | AP3D1  + CSNK2A2 | AP3D1  + HTATSF1 | AP3D1  + CSNK2A2 | CDC40  +  AP3D1 | - | - | - | - | - | - |
