# Supplementary material for: Identification of qPCR reference genes suitable for normalizing gene expression in the mdx mouse model of Duchenne muscular dystrophy
Source: PLoS One. 2019 Jan 30;14(1):e0211384. doi: 10.1371/journal.pone.0211384 (PMC6353192; doi:10.1371/journal.pone.0211384)
Supplement: S7 Table — Pearson correlations (r) for raw Cq values (all genes). Bold: correlations between high scoring candidates (ACTB, RPL13a, CSNK2A2, AP3D1). Italics: correlations with P values greater than 0.0001 (all other correlations P<0.0001); CDC40 vs GAPDH = 0.0026; 18S vs SDHA = 0.0001 (DOCX) [file pone.0211384.s015.docx]

|  | **CDC40** | **AP3D1** | **HTATSF1** | **ACTB** | **FBXW2** | **18S** | **GAPDH** | **PAK1IP1** | **CSNK2A2** | **B2M** | **RPL13A** | **SDHA** | **HPRT1** |
| --- | --- | --- | --- | --- | --- | --- | --- | --- | --- | --- | --- | --- | --- |
| **CDC40** | - | 0.7521 | 0.6118 | 0.6794 | 0.6198 | 0.6556 | *0.2634* | 0.5353 | 0.6943 | 0.5295 | 0.6336 | 0.5833 | 0.6191 |
| **AP3D1** | - | - | 0.6687 | **0.8268** | 0.7812 | 0.7156 | 0.4869 | 0.6078 | **0.8228** | 0.7406 | **0.8276** | 0.5638 | 0.7001 |
| **HTATSF1** | - | - | - | 0.5997 | 0.7891 | 0.5270 | 0.6836 | 0.7151 | 0.7064 | 0.5363 | 0.6462 | 0.7634 | 0.7670 |
| **ACTB** | - | - | - | - | 0.7517 | 0.7293 | 0.4500 | 0.4918 | **0.8646** | 0.7577 | **0.8900** | 0.3712 | 0.6685 |
| **FBXW2** | - | - | - | - | - | 0.7045 | 0.7050 | 0.6958 | 0.7801 | 0.6101 | 0.7631 | 0.5852 | 0.7180 |
| **18S** | - | - | - | - | - | - | 0.4512 | 0.5092 | 0.6735 | 0.4844 | 0.6187 | *0.3417* | 0.4559 |
| **GAPDH** | - | - | - | - | - | - | - | 0.4795 | 0.5056 | 0.4149 | 0.4497 | 0.4209 | 0.4138 |
| **PAK1IP1** | - | - | - | - | - | - | - | - | 0.5328 | 0.4897 | 0.5725 | 0.5936 | 0.6377 |
| **CSNK2A2** | - | - | - | - | - | - | - | - | - | 0.6793 | **0.8515** | 0.5300 | 0.7487 |
| **B2M** | - | - | - | - | - | - | - | - | - | - | 0.7715 | 0.4777 | 0.5993 |
| **RPL13A** | - | - | - | - | - | - | - | - | - | - | - | 0.4120 | 0.7875 |
| **SDHA** | - | - | - | - | - | - | - | - | - | - | - | - | 0.6375 |
| **HPRT1** | - | - | - | - | - | - | - | - | - | - | - | - | - |
