# Supplementary material for: Identification of qPCR reference genes suitable for normalizing gene expression in the mdx mouse model of Duchenne muscular dystrophy
Source: PLoS One. 2019 Jan 30;14(1):e0211384. doi: 10.1371/journal.pone.0211384 (PMC6353192; doi:10.1371/journal.pone.0211384)
Supplement: S8 Table — Spearman’s Rho values for RQ correlations (all genes). Bold: correlations between high scoring candidates (ACTB, RPL13a, CSNK2A2, AP3D1). Italics: correlations with P values greater than 0.0001 (all other correlations P<0.0001); CDC40 vs GAPDH = 0.0046; 18S vs SDHA = 0.0002; GAPDH vs B2M = 0.0003. (DOCX) [file pone.0211384.s016.docx]

|  | **CDC40** | **AP3D1** | **HTATSF1** | **ACTB** | **FBXW2** | **18S** | **GAPDH** | **PAK1IP1** | **CSNK2A2** | **B2M** | **RPL13A** | **SDHA** | **HPRT1** |
| --- | --- | --- | --- | --- | --- | --- | --- | --- | --- | --- | --- | --- | --- |
| **CDC40** | - | 0.7762 | 0.6159 | 0.6567 | 0.6861 | 0.6625 | *0.251* | 0.5246 | 0.7001 | 0.5555 | 0.614 | 0.5688 | 0.6085 |
| **AP3D1** | - | - | 0.6102 | **0.8158** | 0.7742 | 0.6833 | 0.342 | 0.532 | **0.8312** | 0.7608 | **0.8128** | 0.525 | 0.6712 |
| **HTATSF1** | - | - | - | 0.5502 | 0.7773 | 0.5025 | 0.6404 | 0.682 | 0.6797 | 0.4985 | 0.5756 | 0.7669 | 0.768 |
| **ACTB** | - | - | - | - | 0.7403 | 0.6901 | 0.3477 | 0.4195 | **0.8678** | 0.7654 | **0.8714** | 0.3398 | 0.6314 |
| **FBXW2** | - | - | - | - | - | 0.6789 | 0.6046 | 0.7042 | 0.8031 | 0.628 | 0.753 | 0.5818 | 0.7324 |
| **18S** | - | - | - | - | - | - | 0.3941 | 0.4625 | 0.6719 | 0.4769 | 0.5643 | *0.3211* | 0.4374 |
| **GAPDH** | - | - | - | - | - | - | - | 0.4574 | 0.4293 | *0.3176* | 0.3448 | 0.3931 | 0.3764 |
| **PAK1IP1** | - | - | - | - | - | - | - | - | 0.5176 | 0.4227 | 0.4997 | 0.5952 | 0.642 |
| **CSNK2A2** | - | - | - | - | - | - | - | - | - | 0.6949 | **0.8415** | 0.5084 | 0.7364 |
| **B2M** | - | - | - | - | - | - | - | - | - | - | 0.761 | 0.4401 | 0.5986 |
| **RPL13A** | - | - | - | - | - | - | - | - | - | - | - | 0.3614 | 0.7638 |
| **SDHA** | - | - | - | - | - | - | - | - | - | - | - | - | 0.6033 |
| **HPRT1** | - | - | - | - | - | - | - | - | - | - | - | - | - |
